# Somatic mutational landscapes of adherens junctions and their functional consequences in cutaneous melanoma development

**DOI:** 10.7150/thno.46705

**Published:** 2020-10-26

**Authors:** Praveen Kumar Korla, Chih-Chieh Chen, Daniel Esguerra Gracilla, Ming-Tsung Lai, Chih-Mei Chen, Huan Yuan Chen, Tritium Hwang, Shih-Yin Chen, Jim Jinn-Chyuan Sheu

**Affiliations:** 1 Institute of Biomedical Sciences, National Sun Yat-sen University, Kaohsiung 80242, Taiwan.; 2 Institute of Medical Science and Technology, National Sun Yat-sen University, Kaohsiung 80424, Taiwan.; 3 Department of Pathology, Taichung Hospital, Ministry of Health and Welfare, Taichung 40343, Taiwan.; 4 Genetics Center, China Medical University Hospital, Taichung 40447, Taiwan.; 5 Institute of Biomedical Sciences, Academia Sinica, Taipei 11574, Taiwan.; 6 School of Chinese Medicine, China Medical University, Taichung 40402, Taiwan.; 7 Department of Health and Nutrition Biotechnology, Asia University, Taichung 41354, Taiwan.; 8 Department of Biotechnology, Kaohsiung Medical University, Kaohsiung 80708, Taiwan.; 9 Institute of Biopharmaceutical Sciences, National Sun Yat-sen University, Kaohsiung 80242, Taiwan.

**Keywords:** adherens junctions (AJ), cadherin-6 (CDH6), somatic mutation, melanoma, neo-antigen

## Abstract

Cell-cell interaction in skin homeostasis is tightly controlled by adherens junctions (AJs). Alterations in such regulation lead to melanoma development. However, mutations in AJs and their functional consequences are still largely unknown.

**Methods:** Cadherin mutations in skin cutaneous melanoma were identified using sequencing data from TCGA dataset, followed by cross-validation with data from non-TCGA cohorts. Mutations with significant occurrence were subjected to structural prediction using MODELLER and functional protein simulation using GROMACS software. Neo-antigen prediction was carried out using NetMHCpan tool. Cell-based fluorescence reporter assay was used to validate β-catenin activity in the presence of cadherin mutations. Clinical significance was analyzed using datasets from TCGA and other non-TCGA cohorts. Targeted gene exon sequencing and immunofluorescence staining on melanoma tissues were performed to confirm the *in silico* findings.

**Results:** Highly frequent mutations in type-II classical cadherins were found in melanoma with one unique recurrent mutation (S524L) in the fifth domain of CDH6, which potentially destabilizes Ca^2+^-binding and cell-cell contacts. Mutational co-occurrence and physical dynamics analyses placed CDH6 at the center of the top-four mutated cadherins (core CDHs; all type-II), suggesting altered heterophilic interactions in melanoma development. Mutations in the intracellular domains significantly disturbed CDH6/β-catenin complex formation, resulting in β-catenin translocation into cytosol or nucleus and dysregulation of canonical Wnt/β-catenin signaling. Although mutations in core CDH genes correlated with advanced cancer stages and lymph node invasion, the overall and disease-free survival times in those patients were longer in patients with wild-type. Peptide/MHC-I binding affinity predictions confirmed overall increased neo-antigen potentials of mutated cadherins, which associated with T-lymphocyte infiltration and better clinical outcomes after immunotherapy.

**Conclusion:** Changes in cell-cell communications by somatic mutations in AJ cadherins function as one of mechanisms to trigger melanoma development. Certain mutations in AJs may serve as potential neo-antigens which conversely benefit patients for longer survival times.

## Introduction

Cutaneous melanoma is the most dangerous form of skin cancer with high invasive and metastatic potentials, resulting in more than 55,000 deaths per year worldwide 1. Even though the cure rate for patients with localized tumors is high, the overall five-year survival rates could be very low (<20%) for those in whom the spread has occurred, indicating critical roles of cell-cell and cell-matrix crosstalk in disease development. In normal epidermis, melanocytes, or their stem cells, form multiple contacts with keratinocytes, which in turn establish regulatory networks in paracrine manners to fine-tune melanocyte growth and epidermal homeostasis 2, 3. During melanomagenesis, melanoma cells alter such regulatory contacts via extracellular matrix remodeling and then promote dermal invasion by activating inflammation-dependent cell migration/translocation 4. These findings suggest that loss of the control by keratinocytes enables melanocyte undergoing uncontrolled cell growth, leading to cell transformation and melanoma development.

Adherens junctions (AJs) are cadherin-based dynamic structures which link the physical contact from the extracellular matrix to cytoskeleton networks via complex formation between cadherins and catenins (β, α and p120-catenin). Dissociation of β-catenin by growth factor stimulation leads to loss of cadherin-mediated cell-cell contact and increase of β-catenin levels in cytoplasm and nucleus, which subsequently promotes cell proliferation via activating TCF/Wnt signaling 5, 6. On the other hand, AJs also regulate stress-sensing and force-transmission through signaling cascades, known as mechanotransduction 7, 8. Clustering of core cadherin-catenin complexes can further strengthen cell-cell adhesion, allowing cadherins to nucleate signaling hubs and establish the regulatory Hippo network 9, 10. Breakdown of Hippo network by up-regulating expression and nuclear translocation of YAP/TAZ, the nuclear mechanosensor/transducer, was found during melanoma progression, resulting in invasion, metastasis and drug resistance 11-14. Accordingly, AJ-mediated mechanical and cytoskeletal signaling are important for melanoma to create 3D microenvironment for further progression.

Recent studies by using lineage-tracing approach or global gene expression analyses suggested that skin cutaneous melanoma cells may originate from adult melanocyte stem cells and show feature properties of multipotent progenitors 2, 4. Within the specialized anatomical microenvironments, both hemophilic (via type-I cadherins) and heterophilic (via type-II cadherins) cell-cell interactions are required for melanocyte stem cells to develop new cell partnerships with stroma and/or endothelial cells. For examples, cadherin switching from N-cadherin (CDH2) to K-cadherin (CDH6) was found critical for neural crest cell formation, emigration and detachment from the neural tube to be melanocyte 15, 16. Furthermore, down-regulation of key junction proteins for communication between melanocytes and keratinocytes frequently occur during melanoma development, including E-cadherin (CDH1) and P-cadherin (CDH3) 2, 17, 18. Alterations in such interacting network enable melanocyte stem cells to proliferate and migrate out of the niche after activation or transformation by extrinsic stimuli like UV exposure 2, 4.

With advanced whole genome sequencing and other integrative technologies, a set of major driver mutations in cutaneous melanoma, *e.g. BRAF*, *RAS* and *NF1*, have been discovered which contribute to activation of mitogen-activated protein kinase and phosphoinositol kinase signaling 19, 20. Notably, immune signatures with higher lymphocyte infiltration have been correlated with improved survival outcomes but are irrespective of the genomic subtypes in patients 19, 21. These data indicate the need to further explore certain genes that determine microenvironment remodeling and lymphocyte recruitment through cell-cell/cell-matrix interactions. In this study, we aimed to study somatic mutational landscapes of key AJ genes in melanoma and characterize functional impacts of those mutations with molecular and clinical meanings.

## Materials and Methods

### Data collection and functional relevance with mutations in classical cadherin genes

Somatic mutation, gene expression and clinical information data of individual cadherins in skin cutaneous melanoma were collected from cBioPortal databank (https://www.cbioportal.org/) Data from TCGA cohort (TCGA, PanCancer Atlas; n = 448) were utilized as the studying cohort, and the resultant data were validated by using data from non-TCGA cohort (n = 396). The functional interactome among classical cadherins were analyzed by using STRING protein-protein interactome database (http://string-db.org/). Oncoprinter and Mutation mapper tools in cBioportal database were performed to analyze mutation types, hotspot mutation sites, and the conserved mutation sites in cadherin and *CTNNB1* genes 22. To map the key pathways associated with cadherin mutations, normalized gene expression data were applied for gene set enrichment analysis (GSEA) by using GSEA Java (version 2.2.3) from Broad Institute 23. Prediction of peptide-binding affinity onto MHC-I complex, with the peptide length of nine amino acids, was performed to estimate the neo-antigen potentials of mutations in cadherin genes by using NetMHCpan algorithm 24. Mutation-containing peptides with antigen-tolerance score less than 1.0 were considered as neo-antigens.

### Tissue sample collection for targeted exon sequencing and immunofluorescence staining

Twenty-two skin cutaneous melanoma tissue blocks were collected from Department of Pathology, Taichung Hospital, Ministry of Health and Welfare, Taiwan with an approved IRB study (109028) by the Institutional Review Board of Tsaotun Hospital, Ministry of Health and Welfare, Taiwan. Genomic DNA was extracted from those tissue blocks using QIAamp DNA FFPE Tissue Kit (Qiagen, Venlo, Netherlands), followed by PCR enrichment to establish final DNA libraries for targeted exon sequencing (cadherin genes involved in the core interactome) on a HiSeq 2000 (Illumina, San Diego, CA). The raw sequencing data were filtered by Cutadapt to obtain clean data for further quality control checks 25. By using SAMtools, somatic mutations occurred in classic CDHs were identified with an average depth more than 120 counts and an average coverage more than 60% on each chromosome 26. Among the 22 tissues, 9 of them showed mutations in classical cadherin genes in the Taiwanese cohort (Table S1).

For immunofluorescence staining, tissue sections were dewaxed by xylene, followed by alcohol fixation and rehydration. After antigen retrieval in 10 mM citric acid buffer (pH 6.0) and blocking with 5% BSA, tissue sections were incubated with Abs against different core CDHs and co-stained with anti-β-catenin Abs (Cat# MAB14489; Abnova Corp., Taipei, Taiwan) at 4 °C for overnight. The Abs against core CDHs were anti-CDH6 (PAB31388; Abnova Corp.), anti-CDH9 (PA5-106548; Thermo Fisher Scientific Inc., Waltham, MA), anti-CDH10 (ab196662; Abcam PLC., Cambridge, UK) and anti-CDH18 (A14645; Abclonal Inc., Woburn, MA) Abs. For T-lymphocyte infiltration study, anti-CD3 Abs (A19017; Abclonal Inc.) were utilized to co-stain with anti-β-catenin Abs. Secondary Ab staining was carried out by using FITC-conjugated anti-rabbit IgG (for core CDHs and CD3; sc-2359; Santa Cruz Biotech. Inc., Dallas, TX) and CFL555-conjugated anti-mouse IgG (for β-catenin; sc-362267; Santa Cruz Biotech. Inc.) Abs at 37 °C for 1 hour. Auto-fluorescence were quenched using True VIEW auto-fluorescence quenching kit (Vector Lab., Burlingame, CA). Cell nuclei in the tissues were counterstained with DAPI and the images were taken under an IX83 fluorescence microscope (Olympus Corp., Shinjuku, Japan).

### Structural prediction of the cadherin-6 (CDH6) protein

The three-dimensional structures of extracellular cadherin domain 5 (EC5; residues 487-608), juxtramembrane domain (JMD; residues 660-677), and catenin-binding domain (CBD; residues 687-784) of CDH6 protein were predicted by using the MODELLER program (version 9v8) 27. In this study, CDH6 (PDB ID: 5VEB) was selected as the template for predicting the structure of S524L-EC5, p120-catenin in complex with E-cadherin (PDB ID: 3L6X) was selected as template for predicting the structures of WT-, D661N-, and E662K-JMD in complex with p120-catenin, and β-catenin with desmosomal-cadherin (PDB ID: 3IFQ) was selected as the other template for predicting the structures of WT-, R689Q-, D691N-, E722K-, D726N-, S749F-, R773Q-CBD in complex with β-catenin. The qualities of all modeled structures were assessed through Ramachandran plots. The steepest descent algorithm was used for structural optimization, and PyMOL (http://www.pymol.org/) was used for all three-dimensional (3D) structures and charge distribution presentation.

### Molecular dynamics simulations

Molecular dynamics (MD) simulations were performed using GROMACS software (version 5.1.4) 28. The OPLS-AA force field was used for energy calculations. The models were solvated with TIP3P water molecules and simulated in a cubic box with periodic boundary conditions. The energy of the models was first minimized using the steepest descent algorithm. The simulations were performed in the canonical NVT and NPT ensembles through the position-restrained MD simulation for 100 ps, respectively. The linear constraint solver algorithm was used to constrain the bonds. The MD simulations were performed at constant pressure and temperature for 30 ns using an integration time step of 2 fs. The non-bonded interactions were cutoff at 10 Å. The coordinates from the MD simulations were saved every 10 picosecond.

### Binding free energy calculation

The pmx tool was used to construct the hybrid topologies of the system used in the free energy calculations 29. This tool automatically generates hybrid structures and topologies for amino acid mutations that represent the two physical states of the system. The double system (combine both branches of a thermodynamic cycle) in a single simulation box was used as the setup to keep the system neutral at all times during a transition (Figure S1) 29. After obtaining this hybrid structure, free energy simulations were performed using GROMACS. From the 10-ns equilibrated trajectories, 100 snapshots were extracted, and rapid 100-ps simulation was performed starting from each frame. The lambda (λ) ranged from 0 to 1 and from 1 to 0 for the forward and backward integrations, respectively, thus describing the interconversion of the WT to MT systems and the MT to WT systems, respectively. Finally, the pmx tool was used to integrate over the multiple curves and subsequently estimate free energy differences using the fast-growth thermodynamic integration approach, which relies on the Crooks-Gaussian Intersection (CGI) protocol 30. In such a double-system/single-box setup, the estimated free energy corresponds to the ΔΔG of binding (Figure S1).

### Clinical association study and statistical analyses

Clinical data of melanoma patients, including tumor stage, lymph node invasion, metastasis, lymphocyte score and survival time, were retrieved from TCGA database. GraphPad Prism (GraphPad software, La Jolla, CA) was utilized for statistical analyses. Patients were divided into WT CDHs (no mutation in any cadherin genes) and MT core CDHs (patients with mutation in top-four highly-mutated cadherin genes) groups. Statistical differences between two groups were estimated by *t*-test. Kaplan-Meier method was used to estimate survival curves, and a log-rank test was performed for statistical comparison between two groups. To compare neo-antigen potentials, two-way ANOVA was utilized to compare differences between two groups with multiple mutation sites.

### Gene cloning and site-directed mutagenesis

The gene segment of β-catenin was amplified by nested PCR using cDNA from human melanoma A375 cells. After purification, the gene segment was further sub-cloned into pmCherry-N1 vector (Clontech Lab., Madison. WI), thus the gene is tagged with an mCherry at C-terminal end. Human CDH6 cDNA (OriGene Technologies, Inc., USA) was amplified and sub-cloned into pAcGFP1-N1 vector (Clontech Lab.) to generate GFP-tagged wild type CDH6. CDH6 with mutations in the CBD domain were generated by site directed mutagenesis (F541; Thermo Fisher Scientific Inc.). DNA sequencing was done to confirm the gene sequences and the introduced substitutions that are in-frame with the fusion partners.

### Cell-based complex formation assay between β-catenin with CDH6 and its mutant variants

To monitor complex formation in live cells, β-catenin-mCherry and CDH6 variants gagged with GFP were co-transfected into A375 cells using Lipofectamine 2000 (Thermo Fisher Scientific Inc.) by 1:1 molar ratio. Complex formation and cellular localization were determined by an IX83 fluorescent microscope (Olympus Corp., Shinjuku, Japan) 24 hours after gene transfection. Cell image and fluorescent intensity were analyzed using Image-Pro Premier (Media Cybernetics Inc., Rockville, MD).

## Results

### Highly-frequent mutations in adherens junction (AJ) cadherin genes in melanoma

**Figure 1:**
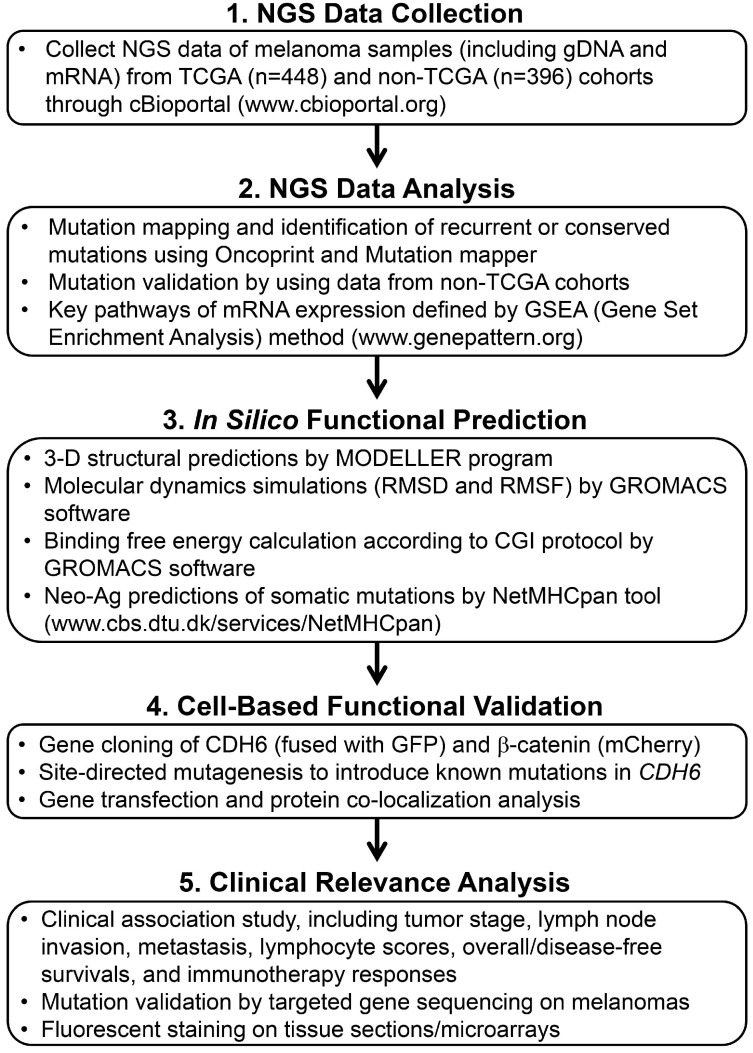
The workflow scheme presents the whole process of data mining and functional characterizations in this study.

**Figure 2:**
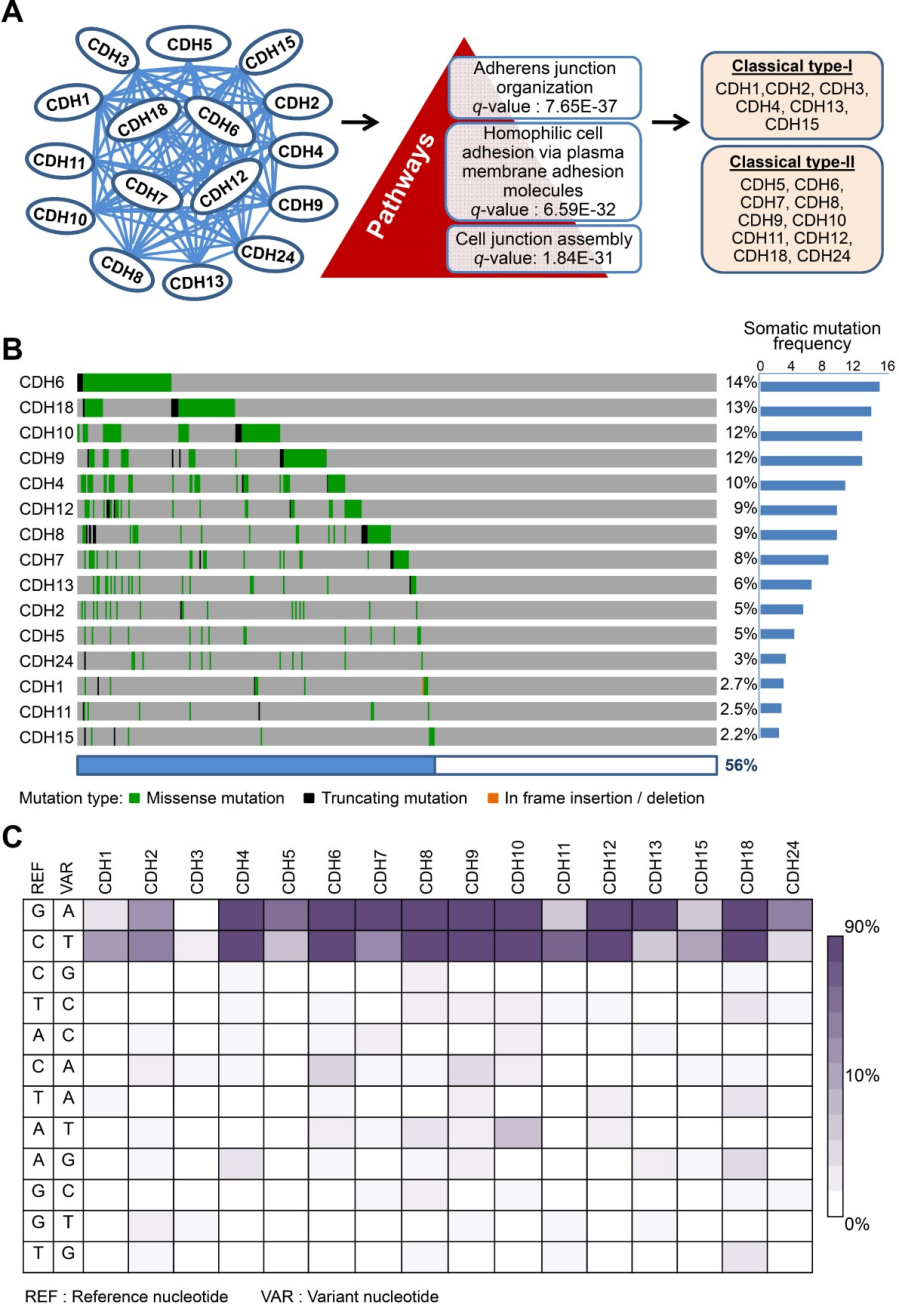
** Mutations in classical cadherin genes in skin cutaneous melanoma.** (**A**) STRING protein-protein interaction analysis (https://string-db.org) on 20 annotated classical cadherins reveals an interactome composed of 16 classical cadherins (6 type-I and 10 type-II) involved in AJ organization, homophilic cell adhesion via plasma membrane adhesion molecules, and cell junction assembly pathways. (**B**) Mutation frequencies of the cadherin genes in skin cutaneous melanoma were analyzed by using NGS data from the TCGA cohort (TCGA, PanCancer Atlas; n = 448). Our data showed no mutation in *CDH3*. (C) The heat map indicates nucleotide substitution profiles of mutated cadherin genes in skin cutaneous melanoma.

Dysregulation in interactions between melanocyte and keratinocyte or melanocyte and matrix is a key step during melanoma malignant transition. We therefore wanted to know mutational profiles of major adhesion molecules, mainly adherens junction (AJ) molecules, in melanoma and their biological impacts on cancer progression (Figure 1). A total of 20 classical cadherins that participate in cell-cell interactions are identified in human genome 31. STRING protein-protein interactome analyses defined a compact network composed by 16 classical cadherins (6 type-I and 10 type-II) that determines AJ organization, homophilic cell adhesion via plasma membrane adhesion molecules, and cell junction assembly with significant scores (*q*-values < 10^-5^) (Figure 2A). TCGA-melanoma data with 448 sequenced tumor samples were utilized to analyze mutation statuses of those 16 genes by using cBioportal databank 32. Surprisingly, frequent mutations (≥ 10%) were found in 5 classical cadherin genes (*CDH6*, *CDH18*, *CDH10*, CDH9 and *CDH4*) (Figure 2B). Although no mutually exclusive pattern, genetic alterations in the classic cadherin genes sum up a total mutation rate of 56% in melanoma tissues. Consistent with previous findings indicating rare E-cadherin (*CDH1*) mutations in cancer 33, only 2.7% of melanomas carry *CDH1* mutations. In addition, no mutation was found in P-cadherin (*CDH3*). Mutation rates in other minor AJ genes including nectins and nectin-like molecules were also relatively low (< 10%) (Figure S2), suggesting that mutations in classical cadherins play more important roles in melanoma development. Such conclusions can be further validated by using sequencing data from non-TCGA cohort, especially for *CDH6*, *CDH9*, and *CDH18* genes which also show mutation rates larger than 10% (Figure S3). Significantly, the top-four mutated genes (core CDHs) are all type-II classical cadherins, suggesting alterations in heterophilic interactions may function as one possible mechanism to drive melanoma development. At nucleotide levels, most of the detected substitutions (around 90%) were G to A or C to T, a signature for DNA damage repair after UV-induced DNA adducts 19 (Figure 2C), indicating the etiological relevance of mutations in those adhesion genes during melanoma development 4.

### Cadherin mutations in melanoma development

**Figure 3:**
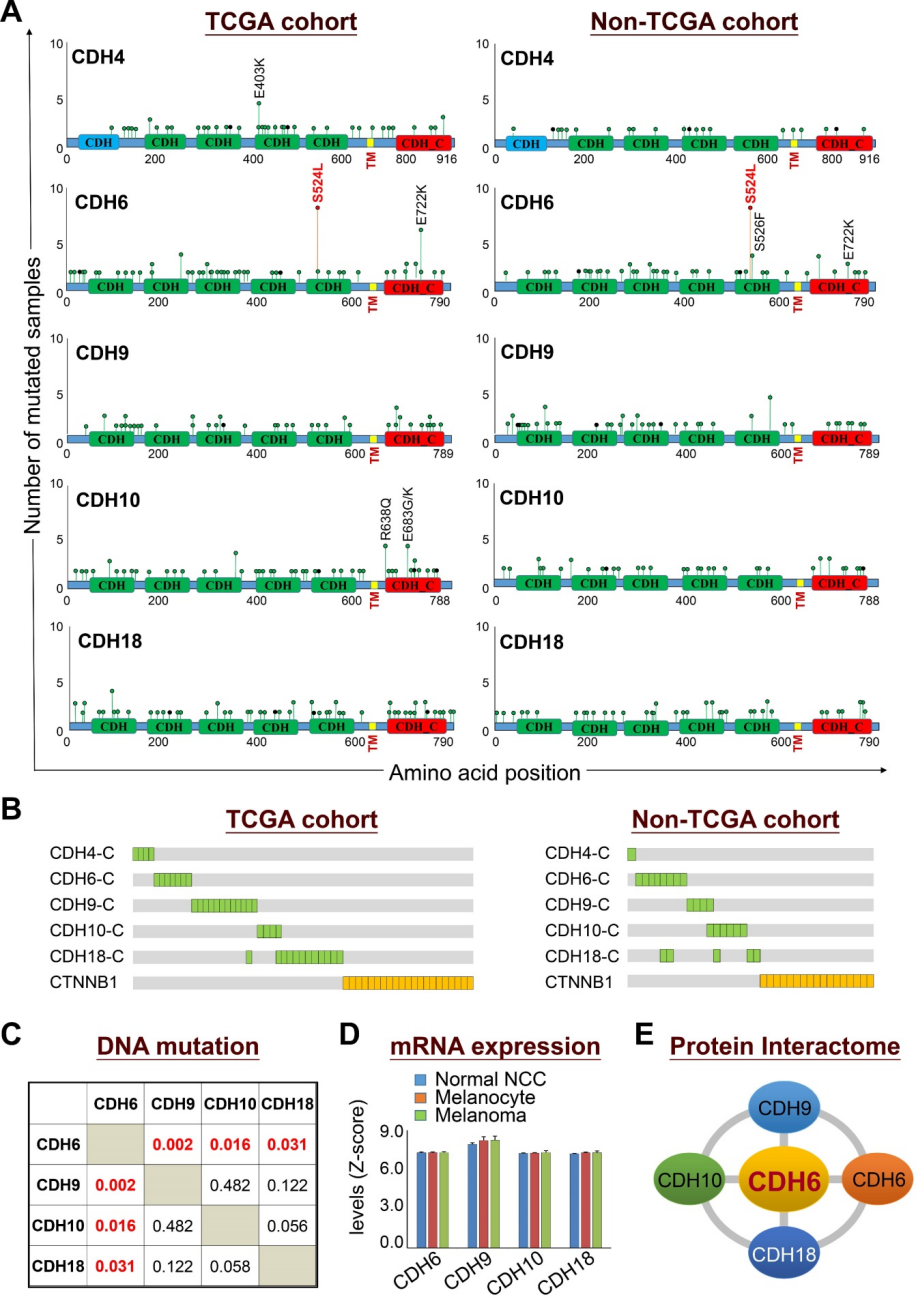
** Biological relevance of mutations in classical cadherin genes in skin cutaneous melanoma.** (**A**) Lollipop plots of mutations in classical cadherin genes in skin cutaneous melanoma were presented by using NGS data from TCGA cohort (n = 448) (*left*) and the non-TCGA cohort (n = 396) (*right*). The non-TCGA cohort includes patients from Broad/Dana Farber (n = 26, Nature 2012), MSKCC (n = 64, NEJM 2014), Broad (n = 121, Cell 2012), Yale (n = 147, Nat Genet 2012), and UCLA (n = 38, Cell 2016). (**B**) Mutations in the intracellular domains of top-five cadherin genes were compared with mutations in *CTNNB1*. (**C**) Co-occurrence analysis among top-four type-II classical cadherin genes (core CDHs) including CDH6, CDH9, CDH10, and CDH18. (**D**) Gene expression levels of core CDHs during melanoma malignant transition from normal neural crest cells and melanocytes to melanoma. Gene expression data were extracted from the GEO dataset (GDS1965). (**E**) Physical interactions (heterophilic/homophilic) among the top-four core CDHs were previously characterized and confirmed 35.

Mutational profiles revealed that somatic mutations in cadherins could be detected at a variety of positions throughout coding regions with the existence of some truncations and out-of-frame insertions/deletions (Figure 3A, *left* panel). The feature of most detected somatic mutations suggests a tumor suppressor role for those adhesion proteins in cancer development 34. Nevertheless, the S524L substitution on the fifth extracellular (EC5) domain of CDH6 (K-cadherin) stood out to be a unique mutation with a relatively higher frequency, indicating its potent role in melanoma development. Especially, this recurrent missense mutation can be confirmed by the non-TCGA cohort (Figure 3A, *right* panel). On the other hand, mutations in the intracellular domains of top-five cadherins show mutual exclusivity with β-catenin (*CTNNB1*) mutations (Figure 3B), suggesting potent effects of cadherin mutations on canonical Wnt/β-catenin signaling.

Co-occurrence analysis among the core CDHs (CDH6, CDH9, CDH10 and CDH10) indicated the association of *CDH6* mutations with mutations in *CDH9*, *CDH10* and *CDH18* (Figure 3C). Gene expression profiling using the GEO dataset (GDS1965) indicated relatively high levels of core CDHs during melanoma malignant transition (Figure 3D). Such expression patterns can be also confirmed at protein levels by immunofluorescence staining (Figures S4-S7). Through biophysical dynamics approach, previous study confirmed strong heterophilic interactions among these core CDHs, and CDH6 was the only one that can form stable hemophilic interactions with other three 35. Interestingly, CDH6 was also the only one to be expressed predominantly in normal melanocytes but not in normal squamous epithelial cells (Figure S4, *upper*). The above data suggested CDH6 as the key player among the core CDHs during melanoma development and progression (Figure 3E). Thus, mutations in type-II cadherins, especially in *CDH6*, may gain functional advantages during melanoma development.

### Structural dynamics of the S524L substitution on the fifth extracellular (EC5) domain of CDH6 (K-cadherin) and the potential impacts on Ca^2+^-binding/stabilization

**Figure 4:**
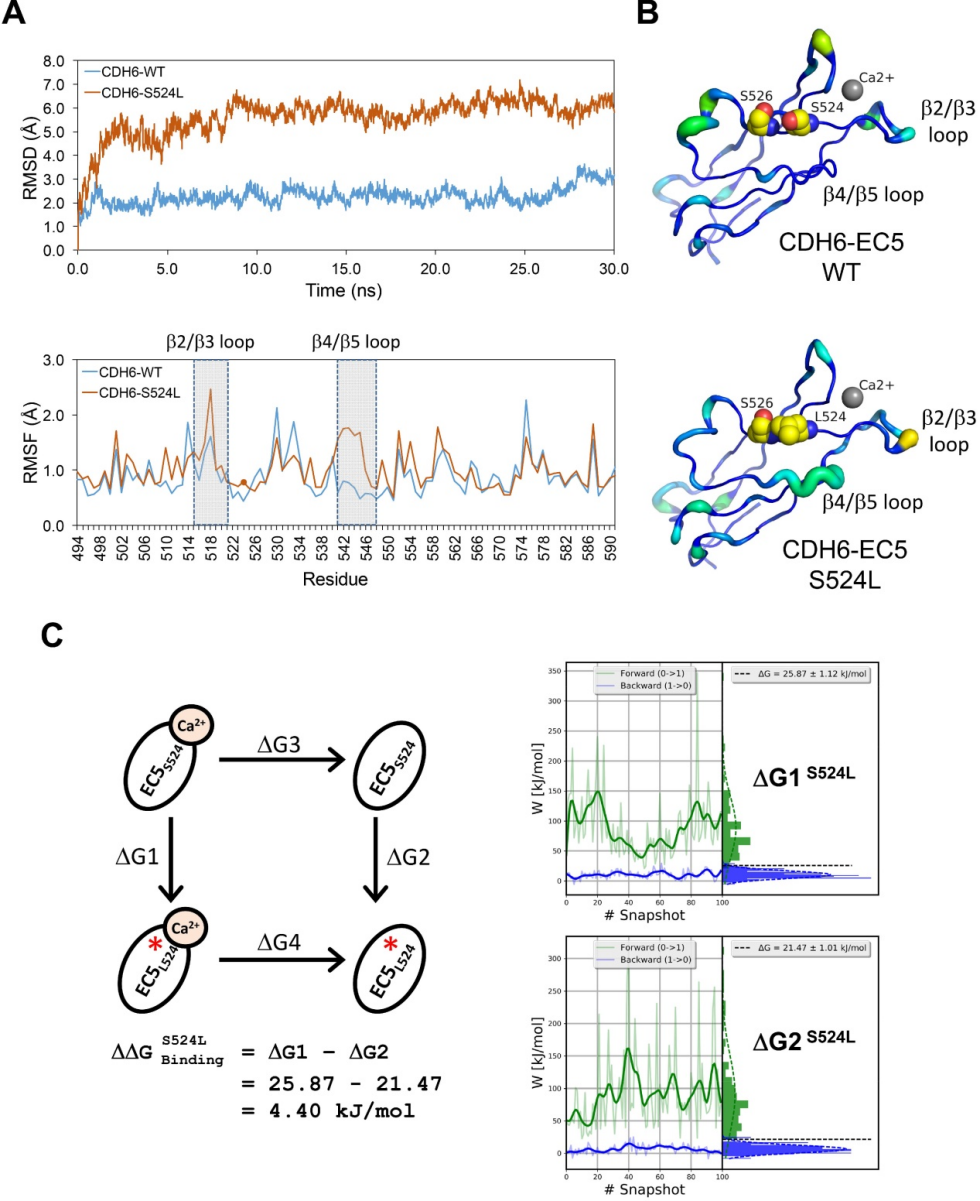
** Impact of S524L mutation on the fifth extracellular (EC5) domain of the CDH6 protein.** (**A**) Protein stability of wild type (WT) and mutant (S524L) CDH6 were compared by residue-specific Root-Mean-Square Deviation (RMSD) and Root-Mean-Square Fluctuations (RMSF) methods in simulations. The S524L mutation lead to higher instability in the β2/β3 loop and β4/β5 loop regions. (**B**) The EC5 structures of the WT and S524L CDH6 are drawn in cartoon putty representation, where the color is ramped by residue from blue as the lowest B-factor value to red as the highest B-factor value. In addition, the size of the tube also reflects the value of the B-factor, where the larger the B-factor the thicker the tube. The calcium atoms and mutation sites are indicated with gray sphere and yellow sphere representations, respectively. (**C**) The thermodynamic cycle for Ca^2+^ binding affinity was calculated in one simulation box. The free energy difference in Ca^2+^ binding corresponds to a double free energy difference: ΔΔG = ΔG1 - ΔG2.

To know functional impacts of mutations in CDH6, the structure of the EC5 domain (CDH6-EC5) was constructed by MODELLER program using the published human CDH6 crystal structure (Protein Data Bank ID: 5VEB) as the template. To compare the similarity in three-dimensional structure of the EC5, Root-Mean-Square Deviation (RMSD) analyses indicated longer average distances between the atoms in the S524L mutant as compared to wild type (WT) CDH6, suggesting its impacts on the EC5 structure (Figure 4A, *upper*). Analyses of the residue-specific Root-Mean-Square Fluctuations (RMSF) (Figure 4A, *lower*) further revealed higher atomic fluctuations of the S524L mutant at the β2/β3 loop, a region for Ca^2+^-binding, when compared with the WT. The β4/β5 loop, which was predicted to stabilize the Ca^2+^-binding, was also found less stable in this mutant due to its higher RMSF and B-factors (Figure 4A-B). To confirm this, free energy differences of Ca^2+^-binding between WT and S524L (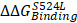) was calculated in a series of alchemical free energy simulations by using CGI protocol 30. The double-free energy was calculated according to Δ*G1* (Ca^2+^-bound) - ΔG2 (Ca^2+^-free), as shown in the thermodynamic cycle (Figure 4C). The data indicated that the Ca^2+^-binding affinity toward CDH6-EC5 was reduced by S524L substitution (ΔΔ*G* = 4.4 kJ/mol). These analyses conclude that S524L substitution may induce additional fluctuations in the β2/β3 and β4/β5 loop regions, which can promote instability in this region and thus hamper Ca^2+^-binding.

### Mutations in the intracellular regions of cadherins (CDH-C) and their effects on catenin-binding

**Figure 5:**
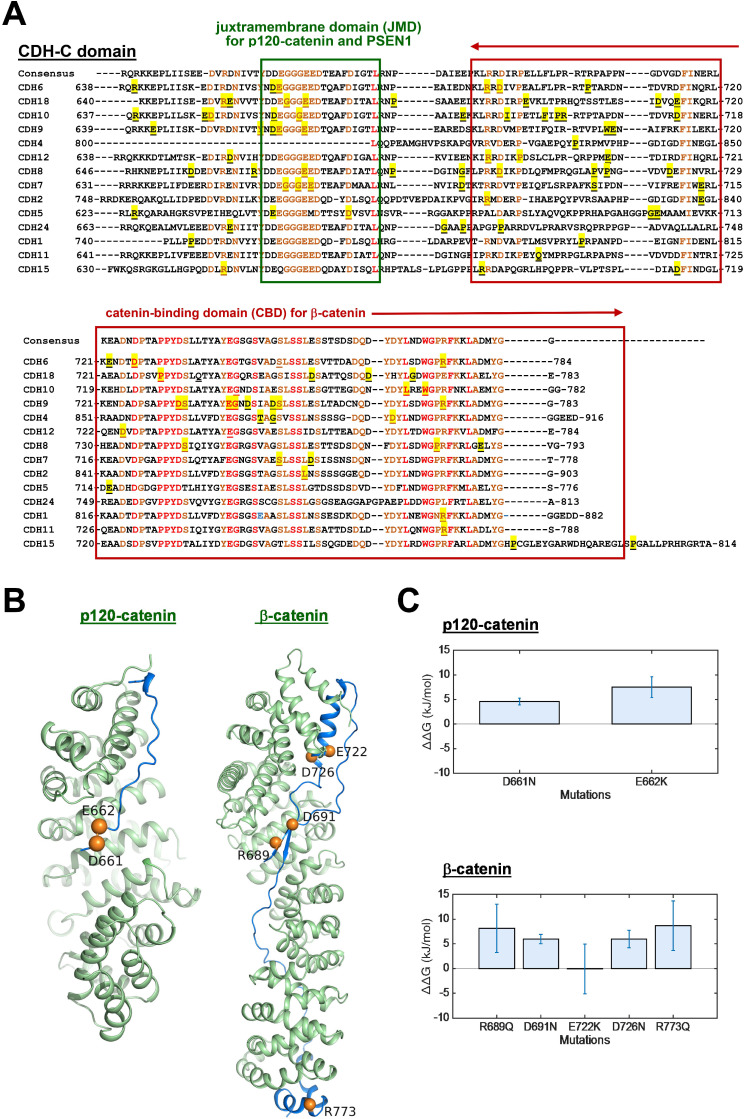
** Thermo-stability of CDH6/catenin complex with mutations in the intracellular domain (CDH-C) of CDH6.** (**A**) Alignment of CDH-C protein sequences among classical cadherins. Letters with underlines and yellow shadows indicate known mutations in the CDH-C sequences in skin cutaneous melanoma based on the NGS data from TCGA cohort. Letters with orange color represent the conserved residues whereas letters in red color represent identical residues among classical cadherins. Green box and red box indicate the regions of JMD for p120-catenin (δ-catenin) and CBD for β-catenin binding, respectively. (**B**) The complex structures of CDH6-JMD (blue)/p120-catenin (green) (*left*) and CDH6-CBD (blue)/β-catenin (green) (*right*) were predicted by using the MODELLER program. The mutation sites are highlighted in orange spheres. (C) The binding free energy differences (ΔΔG) caused by mutations in the domains of JMD for p120-catenin binding (*upper*) and CBD for β-catenin binding (*lower*) were calculated by using the fast-growth thermodynamic integration approach.

The process of AJ assembly to establish cell-cell contacts involves complex formation between cadherins and catenins. To study functional roles of mutations in the intracellular domain (CDH-C), we performed sequence alignment across cadherins involved in the cadherin interactome core. Based on structural and functional analyses, CDH-C can be divided into two major domains: the juxtramembrane domain (JMD) and the catenin-binding domain (CBD) that can bind to p120-catenin (δ-catenin) and β-catenin, respectively (Figure 5A). Most of mutations (>85%) were found involved in the binding regions or conversed residues, indicating potential impacts on cytoplasmic complex formation. We next predicted 3-D structures of CDH6-JMD (residues 660-677) and CDH6-CBD (residues 687-784) in complexes with p120-catenin and β-catenin, respectively, in which orange spheres indicate the mutations (Figure 5B). Significantly, most of the mutations can change the charge (D661N, E662K, R689Q, D691N, E722K, D762N, and R773Q); therefore, the salt bridge interactions between cadherins and catenins can be interrupted, which may consequently reduce cadherin/catenin complex formation.

The cadherin-catenin binding free energy differences between WT and MT structures were calculated by using CGI protocol 30. The catenin binding affinity differences (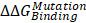) were calculated according to Δ*G1* (catenin-bound) - Δ*G2* (catenin-free), as shown in the thermodynamic cycle when placed in one simulation box (Figure S1A). The net change of the simulation box was considered as zero and negative ΔΔG values indicate stabilizing mutations (Figure S1B). The resulting binding free energy differences (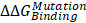) in mutants as compared to WT CDH6 were 4.60 (D661N) and 7.50 (E662K) kJ/mol for p120-catenin (Figure 5C and Figure S8), whereas 8.17 (R689Q), 5.96 (D691N), -0.07 (E722K), 5.98 (D762N), and 8.70 (R773Q) kJ/mol for β-catenin (Figure 5C and Figure S9), suggesting that the binding affinity for both p120-catenin and β-catenin were disrupted in most mutants (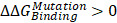). The residue S749 lies within the invariant cadherin sequence 749Ser-Leu-Ser-Ser-Leu753, which has been shown as an important phosphorylation site to form hydrogen bonds with β-catenin 36. The S749F substitution therefore can block the phosphorylation in this region, and consequently reduce cadherin/β-catenin complex formation. Similar strategies were also applied to calculate cadherin-catenin binding free energy differences caused by somatic mutations in other top-four mutated genes. Except CDH10, most substitutions in JMD and CBD domains of those type-II CDHs resulted in less stable cadherin-catenin complexes with increased free energy differences (Table S2).

### Mutations in the CDH-C of CDH6 and their functional consequences

**Figure 6:**
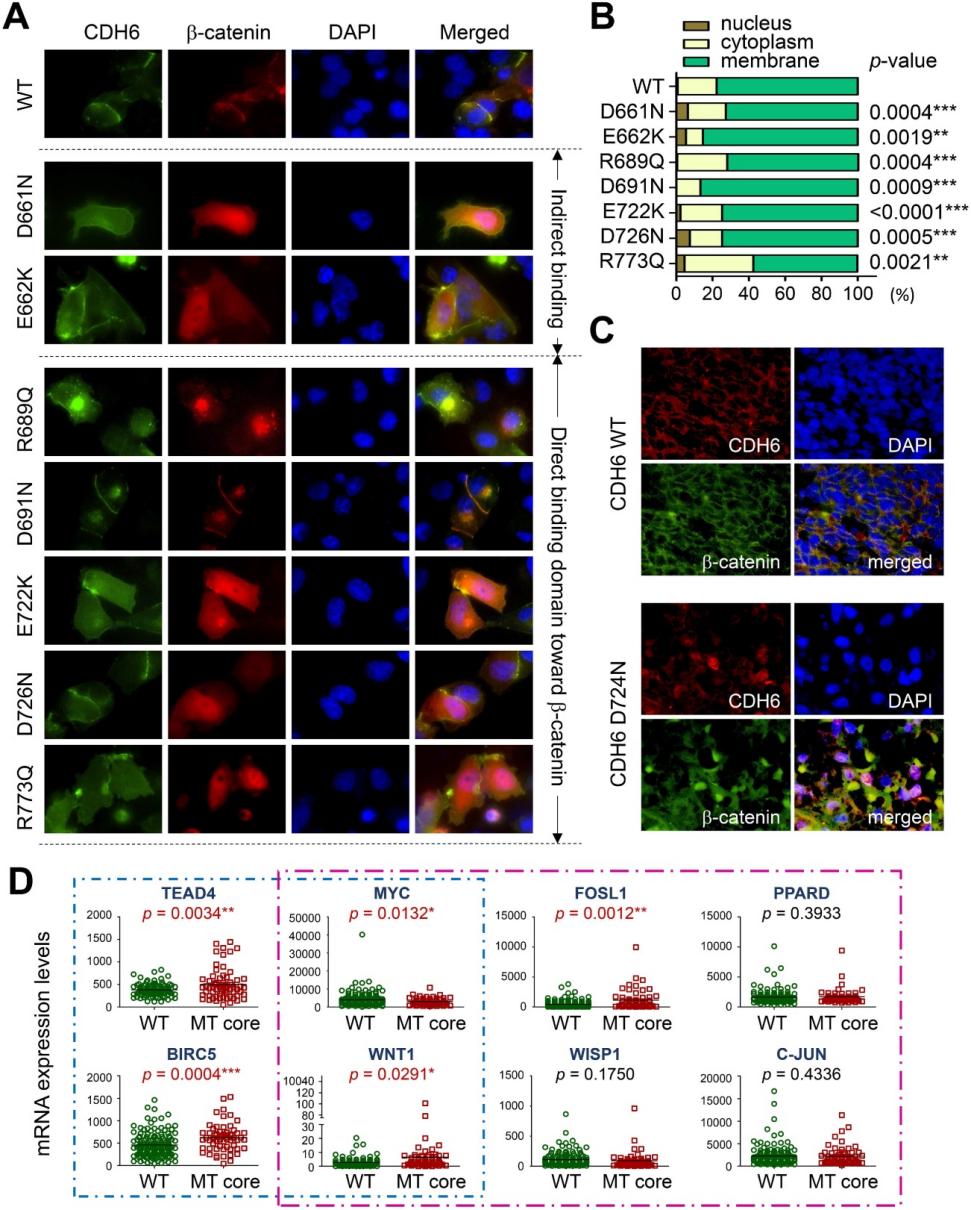
** Functional impacts of mutations in the intracellular domain (CDH-C) of CDH6 on β-catenin binding.** (**A**) Wild type (WT) CDH6 and its mutants (tagged with GFP) were co-transfected with β-catenin (tagged with mCherry) into A375 cells. Cells expressing both green and red constructs were monitored and representative images were taken under a fluorescent microscope 24 hrs after transfection. (**B**) Distributions of β-catenin in cells with different CDH6 constructs were counted and categorized into nucleus, cytoplasm and membrane (n = 50 cells for each CDH6 construct). (**C**) Dual-color immunofluorescence staining, CDH6 in red and β-catenin in green, was performed on melanoma tissue slides from patients with wild type cadherins and patients with D724N mutation in *CDH6*. (**D**) Gene expression levels of genes involved in Hippo (blue box) and Wnt/β-catenin (pink box) pathways were compared between patients with mutations in top-four core cadherins (MT core CDHs) and patients without mutation in any classical cadherins (WT CDHs). The expression levels of indicated genes were extracted from the mRNA data of TCGA cohort. Statistical differences between two groups were compared by using t-test. The *p* values were presented as *: *p* value < 0.05, **: *p* value < 0.01, and ***: *p* value < 0.001.

To study the functional relevance, GFP-tagged CDH6 mutants with different known mutations in CDH-C were prepared individually and co-transfected with wild type β-catenin tagged with mCherry into A375 cells, a melanoma cell line proven to express the lowest CDH6 level in cell line screening. As shown in Figure 6A-B, wild type CDH6 was predominantly expressed on the cell surface in complex with β-catenin. Except D691N, CDH6 mutants with mutations in β-catenin-binding domain significantly reduced complex formation on cell membrane by promoting β-catenin translocation into cytosol and/or nucleus, indicating destabilization of CDH6/β-catenin complex. For D661N and E662K, the portions of nuclear β-catenin were also increased, which could be due to indirect effects of unstable CDH6/p120-catenin complex formation (Figure 6A-B).

To know clinical and biofunctional impacts, we used dual-color immunofluorescence staining to confirm protein expression patterns of core CDHs and β-catenin in clinical samples. For melanoma cells with wild type CDHs, the β-catenin staining usually co-localized with CDH staining on the cell surface. However, nuclear and cytosol β-catenin staining as found in the cell-based study can be frequently detected in melanoma samples with mutated core CDHs (Figure 6C and Figures S4-S7). We also analyzed mRNA levels of genes involved in Hippo and Wnt/β-catenin signaling in patients with mutations in top-four cadherins (MT core CDHs). Patients without any mutation in classical cadherin genes (WT CDHs) were used as the controls. The Hippo-transcription factor TEAD4 was found significantly up-regulated (*p* = 0.0034) in MT core CDHs group which associated with activation of target genes, including BIRC5 (*p* = 0.0004) and WNT1 (*p* = 0.0291) (Figure 6D). The Wnt/β-catenin signaling responsive genes WNT1 (*p* = 0.0291) and FOSL1 (*p* = 0.0012) were also found up-regulated. Coordination among TEAD4, FOSL1, BIRC5 and WNT1 was previously implicated in YAP/Tead-mediated cancer migration/invasion and ion metabolism 37, 38. C-MYC, WISP1 and PPARD were slightly increased but did not reach statistical significance.

### Clinical significance of cadherin mutations during melanoma development

**Figure 7:**
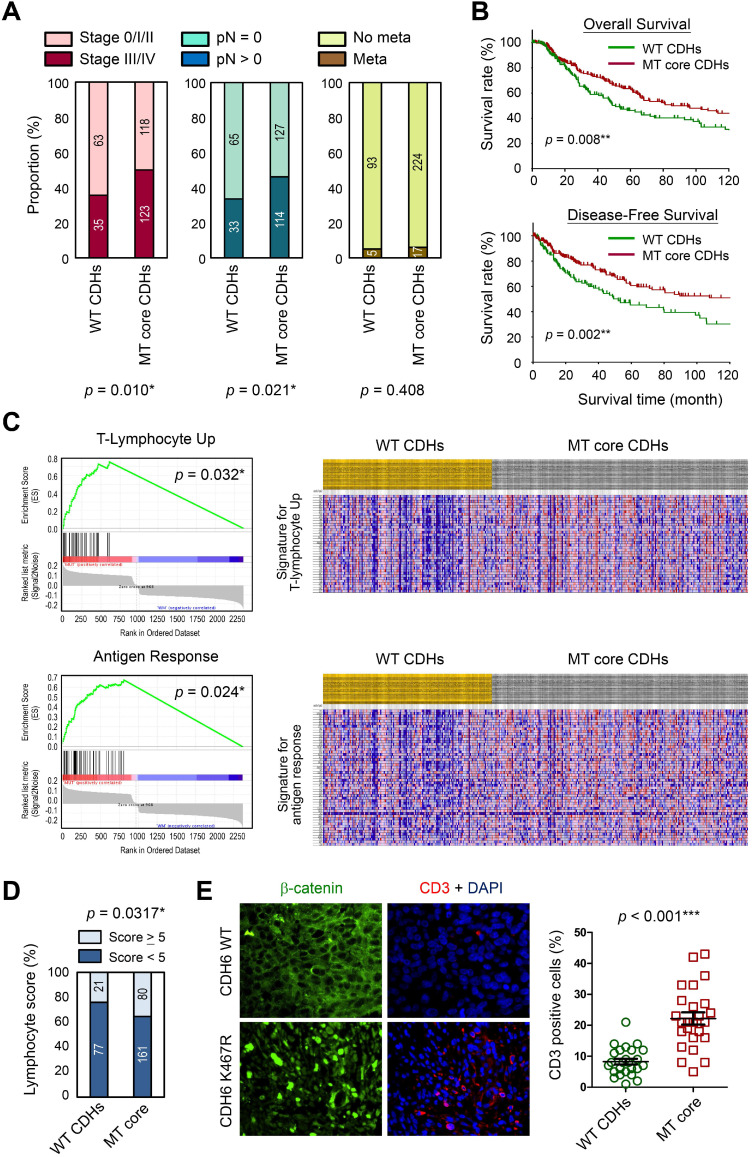
** Clinical significance of cadherin mutations during melanoma development.** (**A**) Clinical associations were compared between patients with mutations in top-four core cadherins (MT core CDHs) and patients without any mutation in classical cadherin genes (WT CDHs), including tumor stage (left), lymph node invasion (*middle*), and metastasis (*right*). (**B**) Kaplan-Meier analyses were performed to compare overall survival (*upper*) and disease-free survival (*lower*) between MT core CDHs and WT CDHs groups. (**C**) Gene set enrichment analysis (GESA) was performed using mRNA expression data from TCGA group. Two pathways, T-lymphocyte-up (*upper*) and antigen response (*lower*) were found positively associated with mutations in top-four core CDH genes. (**D**) Lymphocyte scores in melanoma tissues of TCGA cohort were compared between MT core CDHs and WT CDHs groups. (**E**) Dual-color immunofluorescence staining, CD3 in red and β-catenin in green, was performed on melanoma tissue slides from patients with wild type cadherins and patients with K467R mutation in *CDH6* (*left*). CD3^+^ T-lymphocytes in the stained tissue sections were quantified and compared between MT core CDHs and WT CDHs groups (*right*). For data in (A), (D) and (E), Fisher's exact test was performed to analyze the clinical association. For (B), the long-rank test was used to compare the survival times. The *p* values were presented as *: *p* value < 0.05, **: *p* value < 0.01, and ***: *p* value < 0.001.

To address the translational relevance, we performed clinical association study by subgrouping patients into MT core CDHs and WT CDHs groups. Our data revealed that patients in the MT group are at higher risks to get more aggressive tumors at advanced stages (stage III/IV) (*p* = 0.010; Figure 7A, *left*) and lymph node invasion (*p* = 0.021; Figure 7A, *middle*). Although we found higher tendency of metastasis for the MT group (6.2 % *vs.* 5.1 %; Figure 7A, *right*), the data did not reach statistical significance. Surprisingly, such tumor-promoting activity did not determine worse survival outcomes. In general, patients with mutations in core CDH genes showed better overall (*p* = 0.008; Figure 7B, *upper*) and disease-free (*p* = 0.002; Figure 7B, *lower*) survival times. To know possible signaling signatures in melanomas with mutations in core CDH genes, gene set enrichment analysis (GSEA) was performed using WT group as the control. Several up-regulated pathways were found to contribute to cancer progression, DNA replication/cell proliferation, and cell cycle regulation (Table S3 and Figure S10), indicating the pro-oncogenic activity of cadherin mutations. These data may explain why melanomas with cadherin mutations correlate with advanced tumor stages and metastatic potentials.

Interestingly, T-lymphocyte-up and antigen response pathways were also found to be activated in MT group (Figure 7C), suggesting that anti-tumor immune responses may be triggered once tumor cells enter the circulation system. Consistent with those findings, patients in MT group showed higher lymphocyte scores (including lymphocyte density and distribution) in their tumor lesions as compared to patients in the control group (Figure 7D). To confirm such finding in real clinical samples, melanoma tissue sections with matched genetic information (Table S1) were utilized to detect T-lymphocytes (CD3^+^; red) in melanomas. Our data revealed higher T-lymphocyte (CD3^+^) infiltrations in melanoma lesions with mutations in core CDH genes as compared to tissues with wild type CDHs (Figure 7E). Our data suggest that cadherin mutations can potentially trigger anti-cancer immunity in patients via promoting lymphocyte infiltration, resulting in prolonged survival times.

### Cadherin mutations generate neo-antigens that favor better immunotherapy outcomes

**Figure 8:**
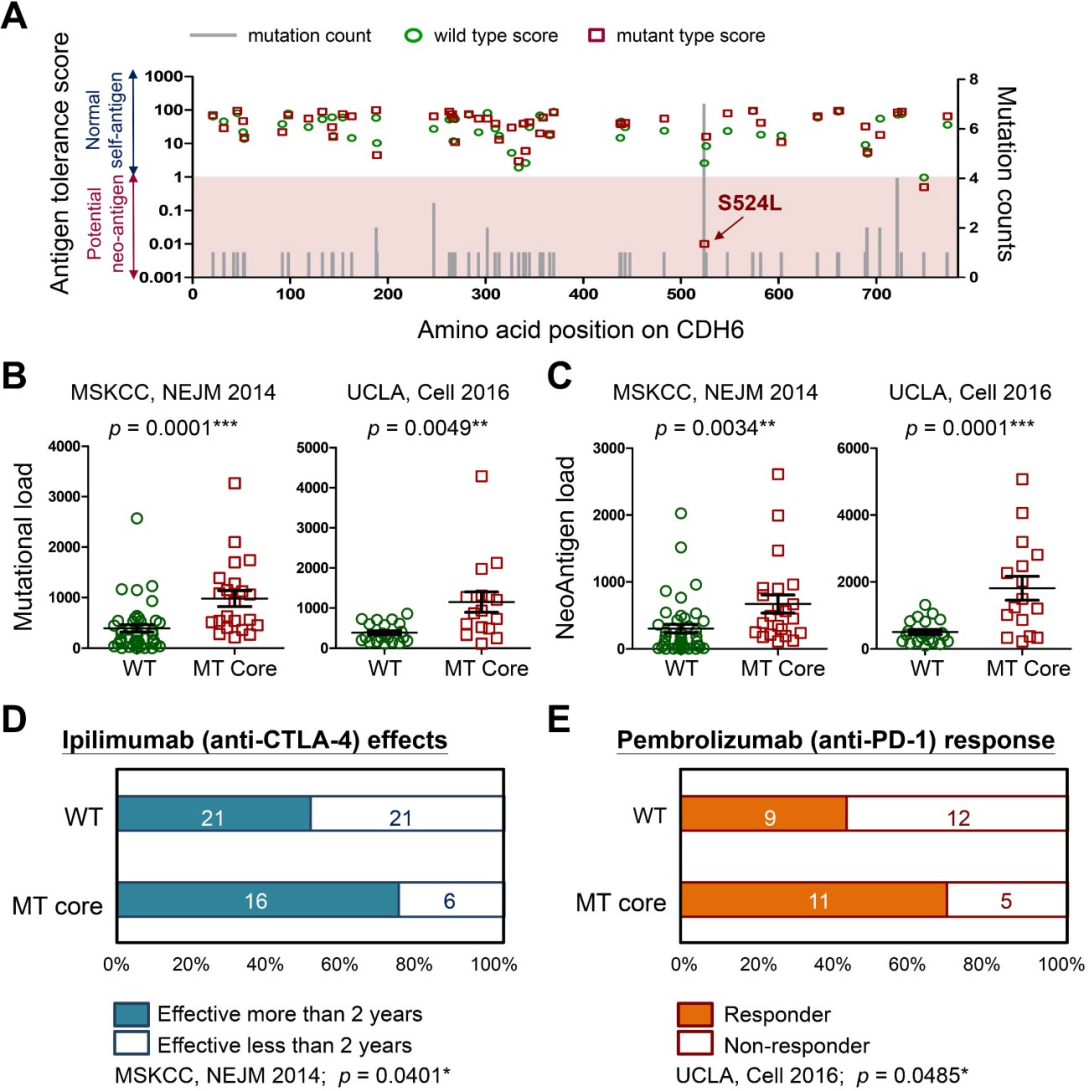
** Immunogenicity of cadherin mutations and therapeutic advantages for treating melanoma.** (**A**) The mutation counts (gray bars) for known *CDH6* mutations in TCGA cohort were shown alone with the amino acid positions in CDH6. Antigen tolerance scores of the indicated mutation (red square) and its corresponding wild type (green circle) were analyzed by using NetMHCpan algorithm and plotted on this bar chart. Immunogenetic datasets of MSKCC 39 and UCLA 41 were utilized to compare (**B**) overall mutational loads; (**C**) Neo-antigen loads; (**D**) therapeutic duration times for Ipilimumab (anti-CTLA-4) treatment; and (**E**) drug responses for Pembrolizumab (anti-PD-1) therapy between MT core CDHs and WT CDHs groups. For data in (B) and (C), Fisher's exact test was performed to analyze the statistical significances. For (D) and (E), two-proportions Z-Test was utilized to compare drug effectiveness. The *p* values were presented as *: *p* value < 0.05, **: *p* value < 0.01, and ***: *p* value < 0.001.

To understand how cadherin mutations activate T-cell immunity, we used machine-learning-based neo-antigen prediction program, NetMHCpan 24, to score neo-antigen potentials of defined mutations. Notably, the recurrent S524L substitution on CDH6 was found to be a potent neo-antigen due to increased MHC-I binding affinity and less antigen-tolerance (Figure 8A). Predictions on other three core cadherins confirmed an overall decrease of antigen-tolerance scores associated with known somatic mutations (Figure S11). This finding indicated higher immunogenic potentials generated by mutations in core CDH genes. Accumulating evidence have revealed that tumors with higher mutational burdens correlate with the likelihood of higher neo-antigen loads, leading to better therapeutic outcome for patients after immunotherapy 39, 40. By using immunogenetic datasets from MSKCC and UCLA groups 39, 41, we found that mutations in core CDH genes highly correlated with total mutational and neo-antigen loads in patients with metastatic melanoma (Figure 8B-C). Those findings suggest an anti-tumor microenvironment immune type associated with mutations in core CDH genes that favors a better clinical response to immunotherapeutic agents (Figure S12) 42. To confirm this, we also compared clinical outcomes of anti-CTLA4 (Ipilimumab) 39 and anti-PD1 (Pembrolizumab) 41 therapies between patients with and without mutations in core CDH genes. Consistent with higher mutational and neo-antigen loads, patients with CDH mutations usually showed longer therapeutic duration times for Ipilimumab treatment (Figure 8D) and better drug responses for Pembrolizumab therapy (Figure 8E).

## Discussion

Alterations in cell-cell contacts have long been considered as an important determinant of cancer initiation and progression. Although molecular bases of many well-known cadherins *e.g.* CDH1 (E-cadherin) and CDH2 (N-cadherin) in cancer development have been established, we know very little about genetic/functional impacts of other AJ genes on cancer especially cutaneous melanoma, a unique cancer type with a high mutation load. Through data mining using TCGA dataset, we discovered high mutation frequencies in cadherin genes which can be validated by using data from non-TCGA cohort (Figure 2 and Figure S3A). Interestingly, we found the top-four highly mutated genes (core CDHs, including *CDH6*, *CDH18*, *CDH10* and *CDH9*) are all type-II cadherins which harbor the capability to form heterophilic interactions. By contrast, mutation frequencies in type-I cadherins, which form homophilic interactions, were relatively low. Even though mutation rate of CDH4 (R-cadherin) was also high (the fifth highly mutated gene) and defined as a type-I cadherin, the heterophilic interaction between CDH4 and CDH2 has been previously reported 43. Dislike CDH1 being genetically deleted or epigenetically silenced during cancer development, those top-four cadherins were consistently expressed to form cell-cell contacts during the transition from melanocyte to melanoma. Our study therefore provides genetic evidence to support the view that the heterophilic interactions between different cell types, such as melanocytes with keratinocytes or stroma cells, play critical roles in skin tissue homeostasis. Breaking such balance by mutations in key cadherins may serve as a newly-defined driving force to promote melanomagenesis, which could be a possible common mechanism shared by other cancer types. Pan-cancer study of AJ genetic alterations is under investigation.

Among AJ genes in cutaneous melanoma, CDH6 stood out to be a very interesting one which showed the highest mutation rate with several novel functional mutations. Through cadherin binding dynamics assays 35, CDH6 was also found at the center of the interactome formed by the top-four core CDHs. Previous study has shown CDH6 a node protein for the interplay of multiple cellular signaling such as TGF-β, PI3K/AKT and FAK/ERK1/2, thus participates in the regulation of stem cell formation and function 44, 45. Both tumor-suppressing 46, 47 and pro-oncogenic activities 45, 48, 49 were associated with CDH6 expression in cancer development. Emerging evidence in recent years suggest a pro-EMT role of CDH6 to promote cancer invasion and metastasis 48, 49. Those controversial results signify complex regulation in inter-/intra-tissue crosstalk during cancer development and progression. Based on our study, recurrent mutation S524L and functional mutations in the intracellular domain of CDH6 can result in loss of cell-cell contacts and β-catenin translocation into nucleus, which associate with tumors at more advanced stages and lymph node invasion (Figures 6 and 7), suggesting those mutations as active drivers but not passengers during melanogenesis. To our knowledge, this is the first report to address the functional impacts of CDH6 and its mutations on melanoma development. Interestingly, we also found nuclear staining for mutated CDH6 in tumor samples (Figure 6C), which could be novel phenomena in certain types of melanoma awaiting further investigation.

Type II cadherins have been shown to exhibit either homophilic or heterophilic binding capability in cell aggregation studies 50-52 and their interactions define the development of nervous system in vivo 51, 53, 54. Expression of specific complements of type II cadherins in neuron cells can determine their cell sorting during embryonic development into distinct sub-populations with different phenotypes/functions 55. It has been known that melanocytes as well as their stem cells in the skin originate from neural crest cells migrating out along the dorsal neural tube. In this process, changing cell-cell contacts by cadherin switching plays important roles to trigger cell de-polarization and epithelial-mesenchymal transition, leading to cell detachment and migration 56, 57. The well-known type-II cadherins involved in the underlying mechanisms include CDH6 15, CDH7 58, and CDH11 59. Recent studies on biophysics of collective cell migration revealed that cancer cell metastasis is a process very similar to neural crest cell spreading 60-62. This viewpoint highlights the requirement of changes in heterotypic mechanical interactions for cells to pass through the mechanical barriers generated by primary cell-cell contacts in the original tissue. For examples, fibroblast-like synoviocytes can migrate and invade into pigmented villonodular synovitis by expressing CDH11 63, Silencing of CDH10 was also found as another mechanism to enhance breast tumor cell mobility in hypoxic conditions 64. These studies support our findings that mutations in core type-II cadherins may provide advantages for melanoma cells to alter the original connecting networks with keratinocytes, enabling them to further migrate out of melanocyte stem cell niche and form tumor lesions on the skin.

Based on genetic analyses, molecular modeling and experimental investigations, our data highly suggest the causal consequence of mutations in CDH-C domains with activation of Wnt/β-catenin signaling. Firstly, mutations in CDH-C domains of the core CDHs showed mutual exclusivity with β-catenin (*CTNNB1*) mutations (Figure 3B), suggesting that they share redundant biological functions; secondly, CDH-C mutations correlated with increased thermodynamics of the CDH-C/β-catenin or CDH-C/p120-catenin complexes (Figure 5C and Figures S8-S9), indicating higher levels of free β-catenin in cells; finally, live imaging study confirmed an overall increased nuclear β-catenin in cells expressing CDH6 mutants with mutations in the CDH-C domain (Figure 6A-B). Consistent with this, the recurrent S524L mutation in CDH6-EC5 also affect the stability and organization of CDH6-mediated AJ complex (Figure 4A-C), leading to β-catenin release from the membrane and translocation into cell nucleus as well as loss of contact inhibition (Figure 6B-D). Especially, there is a 510-His-Ala-Val-512 (HAV) motif at the EC5 domain which is considered responsible for stabilization and clustering of adjacent cadherin monomers in a calcium-dependent fashion 65, 66. Also, the EC5 is the only extracellular domain that contains critical cysteine residues (at the positions of 497, 589, 591, and 600) in CDH6 for possible cis- and trans-dimerization of neighboring cadherins. Therefore, S524L mutation may restrain calcium-induced CDH6 rigidification and dimerization, leading to reduced cell contacts with neighboring cells.

Although mutations in the core CDH genes showed pro-oncogenic activity, patients with such mutations showed better overall and disease-free survival times (Figure 7B). These results indicate possible complications associated with mutations in those type-II cadherins in controlling immunosurveillance. Several cadherins including CDH6 were identified as RGD motif-containing proteins, which can function as ligands to activate metastasis-associated integrins, including β1-containing integrins such as α2β1, and promote cancer cell proliferation, migration and invasion 67-69. Signaling activated by those metastasis-associated integrins can also enhance tumor angiogenesis 68-70. By contrast, activation of β1-containing integrins in γδT cells, a specialized skin-resident/infiltrating T cells, can also promote cell adhesion to endothelial cells, extravasation and lymphocyte infiltration into tumors 71-73. γδT cells trigger immune response rapidly just like innate-like lymphocytes do, yet they can direct the development of adaptive immunity with TCR-dependent reactivity/specificity 74, 75. By using the immunogenetic dataset from MSKCC group 39, we also noticed patients with mutations in *CDH6* or other core CDH genes showed more γδT cell recruitments in melanoma lesions with more activated CD4^+^ memory T cells and higher cytolytic scores (Figure S12). Accordingly, there could be a possibility that mutations in cadherins, when the melanoma cells spread through the blood or lymph, can stimulate γδT cell activation and enhance lymphocyte recruitment into core tumor lesions, leading to up-regulation of T-lymphocyte and antigen responses which were evidenced in our study (Figure 7C-E).

A recent study indicated increased hydrophobicity of UV-derived mutations that can enhance antigen presentation on the MHC complexes with stronger neo-antigen potentials 76. Since most of detected CDH mutations in melanoma are highly relevant to UV exposure, CDH mutations should play certain key roles in triggering anti-tumor immunity in patients. Especially, the S524L substitution in CDH6 has been predicted to be a strong neo-antigen (Figure 8A), peptides with such substitution could be utilized as potent adjuvants to trigger stronger anti-melanoma immunity when co-treated with other immuno-therapeutics such as anti-PD-1/L1 or anti-CTLA4 Abs. Whether mutations in cadherins can change cytokine profiles in the local microenvironment to direct lymphocyte homing and activate anti-melanoma immunity will be investigated in our future study.

Some limitations in this study need to be addressed here. Firstly, the mutational profiles detected in the Taiwanese cohort were found a little bit different from the TCGA cohort which is Caucasian dominant (96%) 19. These differences suggest the involvement of genetic factors between different populations during melanomagenesis. However, CDH6, CDH10, CDH18 are still within the top-five most frequently mutated cadherin genes in the Taiwanese cohort (Table S1). Also, most of detected mutations are related to UV-exposure (G to A or C to T substitutions). Therefore, protein staining data by using Taiwanese samples might be relevant but mutation sites are different. Tumor samples from Caucasian population with matched genetic information may provide the opportunity to confirm biological effects of those mutations shown in Figure 6A-B. Secondly, CDH mutations alone may not be the only contributor to make melanoma becoming more immunogenic. Functional impacts from other genetic events during melanoma development should be also considered and investigated.

## Supplementary Material

Supplementary figures and tables.

## Abbreviations

AJs: adherens junctions
CDH: cadherin
core CDHs: the top-four mutated cadherins including CDH6, CDH9, CDH10 and CDH18
Neo-Ag: neo-antigen
GSEA: gene set enrichment analysis
EC5: extracellular cadherin domain 5
JMD: juxtramembrane domain
CBD: catenin-binding domain
MD: molecular dynamics
WT: wild type
MT: mutant type
ΔΔG: free energy differences
