# Supplementary material for: Somatic mutational landscapes of adherens junctions and their functional consequences in cutaneous melanoma development
Source: Theranostics. 2020 Oct 26;10(26):12026–43. doi: 10.7150/thno.46705 (PMC7667680; doi:10.7150/thno.46705)
Supplement: Supplementary file 1 — Supplementary figures and tables. [file thnov10p12026s1.pdf]

**Table S1.** Genetic mutation rates of core CDH genes based on targeted exon sequencing in melanoma samples from Taiwanese patients

| **Gene** | **Extracellular domain and transmembrane mutation rate (%)** | **Substitution events[a]** | **Intracellular domain mutation rate (%)** | **Substitution events[a]** | **Total mutation rate (%) (cases/ total samples)** |
|---|---|---|---|---|---|
| CDH6 | 9.09% | exon9:c.A1400G:p.K467R | 18.18% | exon12:c.G2170A:p.D724N<br>exon12:c.G2236A:p.V746M | 22.73%<br>(5/22) |
| CDH10 | 18.18% | exon3:c.C346G:p.R116G<br>exon3:c.C377T:p.T126I<br>exon5:c.G607A:p.E203K<br>exon7:c.C1163T:p.S388F | 9.09% | exon12:c.G2044A:p.E682K | 22.73%<br>(5/22) |
| CDH18 | 13.63% | exon4:c.G310A:p.D104N<br>exon8:c.G1124A:p.G375E<br>exon9:c.G455A:p.G152E | 13.63% | exon16:c.G2345A:p.G782E | 22.73%<br>(5/22) |
| CDH12 | 18.18% | exon6:c.C28T:p.L10F<br>exon6:c.C65T:p.P22L<br>exon12:c.T1301C:p.I434T<br>exon15:c.G1802A:p.C601Y<br>exon16:c.C2186T:p.T729I | 0% | None detected | 18.18%<br>(4/22) |
| CDH9 | 9.09% | exon2:c.C62T:p.T21I<br>exon4:c.G541A:p.V181I | 0% | None Detected | 13.63%<br>(3/22) |
| CDH24 | 9.09% | exon3:c.C395T:p.P132L<br>exon3:c.C470T:p.A157V | 4.54% | exon13:c.G2036A:p.R679Q | 13.63%<br>(3/22) |

| | | | | | |
|---|---|---|---|---|---|
| | | exon8:c.G1279A:p.E427K | | | |
| CDH1 | 9.09% | exon8:c.C1082T:p.A361V<br>exon10:c.G1483A:p.V495M | 4.54 | exon14:c.G2287A:p.E763K | 9.09%<br>(2/22) |
| CDH2 | 4.54% | exon9:c.G1276A:p.D426N<br>exon11:c.C1725A:p.F575L | 9.09% | exon15:c.G2476A:p.A826T<br>exon16:c.T2593G:p.S865A | 9.09%<br>(2/22) |
| CDH4 | 9.09% | exon1:c.C44T:p.P15L<br>exon14:c.A2338C:p.I780L | 0% | None detected | 9.09%<br>(2/22) |
| CDH5 | 9.09% | exon4:c.C556T:p.H186Y<br>exon11:c.G1798A:p.A600T | 0% | None detected | 9.09%<br>(2/22) |
| CDH7 | 9.09% | exon3:c.G433A:p.D145N<br>exon11:c.G1789A:p.E597K<br>exon11:c.G1864A:p.V622M | 4.54% | exon12:c.C2264T:p.S755F | 9.09%<br>(2/22) |
| CDH8 | 9.09% | exon3:c.G340A:p.V114I<br>exon3:c.C445G:p.P149A<br>exon10:c.A1589C:p.Y530S | 0% | None detected | 9.09%<br>(2/22) |
| CDH11 | 9.09% | exon3:c.C65T:p.A22V<br>exon5:c.G607A:p.E203K | 0% | None detected | 9.09%<br>(2/22) |
| CDH13 | 4.54% | exon5:c.C557T:p.S186F<br>exon6:c.G760A:p.V254I | 9.09% | exon14:c.C2143T:p.P715S<br>exon14:c.G2170A:p.V724I | 9.09%<br>(2/22) |
| CDH15 | 4.54% | exon9:c.G1306A:p.V436M | 0% | Not detected | 4.54%<br>(1/22) |

[a]c- chromosomal base substitution; p- amino acid substitution

**Table S2.** Calculation of catenin-binding free energy differences between WT and known mutations in core CDH genes in melanoma

| Mutation | Protein | Domain | Binding protein | $\Delta\Delta G_{Binding}^{Mutation}$ (kJ/mol) |
|---|---|---|---|---|
| D661N | CDH6, CDH9, CDH10 | JMD | p120-catenin | 4.60 ± 0.69 |
| E662K | CDH6, CDH9 | JMD | p120-catenin | 7.50 ± 2.11 |
| G663E | CDH18 | JMD | p120-catenin | 42.29 ± 7.57 |
| G665R | CDH10 | JMD | p120-catenin | -15.34 ± 13.85 |
| E666K | CDH9 | JMD | p120-catenin | 21.95 ± 4.39 |
| E667K | CDH18 | JMD | p120-catenin | 1.56 ± 2.88 |
| R689Q | CDH6 | CBD | β-catenin | 8.17 ± 4.91 |
| D691N | CDH6 | CBD | β-catenin | 5.96 ± 0.96 |
| E722K | CDH6 | CBD | β-catenin | -0.07 ± 5.04 |
| D726N | CDH6 | CBD | β-catenin | 5.98 ± 1.77 |
| D733N | CDH9 | CBD | β-catenin | 15.62 ± 1.83 |
| E741K | CDH9 | CBD | β-catenin | 16.38 ± 2.35 |
| G742E | CDH10 | CBD | β-catenin | -19.63 ± 6.25 |
| L767F | CDH10 | CBD | β-catenin | -1.60 ± 5.56 |
| R773Q | CDH6, CDH9 | CBD | β-catenin | 8.70 ± 5.05 |

**Table S3.** Gene set enrichment analyses of reactomes/pathways concurrently up-regulated with mutations in top-four core CDH genes in melanoma[a]

| Reactome /Pathway[b] | ES | NES | NOM *p*-value |
|---|---|---|---|
| 1. Liver_Cancer_Subclass_G3_Up | 0.6493 | 1.7587 | 0.0159 |
| 2. Doxorubicin_Resistance_Up | 0.7836 | 1.6533 | 0.0175 |
| 3. Antigen_Response | 0.6726 | 1.7603 | 0.0236 |
| 4. Thyroid_Carcinoma_Anaplastic_Up | 0.5938 | 1.7737 | 0.0276 |
| 5. T_Lymphocyte_Up | 0.7554 | 1.6582 | 0.0320 |
| 6. Epithelial_Mesenchymal_Transition_Up | 0.6731 | 1.7110 | 0.0333 |
| 7. M_G1_Transition | 0.6640 | 1.6491 | 0.0345 |
| 8. Core_Serum_Response_Up | 0.6037 | 1.6801 | 0.0352 |
| 9. Cell_Cycle_Mitotic | 0.6362 | 1.6307 | 0.0369 |
| 10. Synthesis_Of_DNA | 0.6648 | 1.6498 | 0.0381 |
| 11. Mitotic_G1/G1_S_Phases | 0.5985 | 1.6321 | 0.0386 |
| 12. CDC20_Mediated_Degradation_Of_Mitotic_Proteins | 0.6666 | 1.6343 | 0.0423 |
| 13. DNA_Replication | 0.6606 | 1.6724 | 0.0437 |
| 14. Mitotic_M/M_G1_Phases | 0.6589 | 1.6693 | 0.0437 |
| 15. Cervical_Cancer_Proliferation_Cluster | 0.6992 | 1.6660 | 0.0483 |

[a]Abbreviations: ES, enrichment score; NES, normalized enrichment score; NOM *p*-value, nominalized *p*-value.

[b]Reactomes or pathways with NOM *p*-values $< 0.05$ were considered as significantly relevant with mutations in top-four core CDH genes.

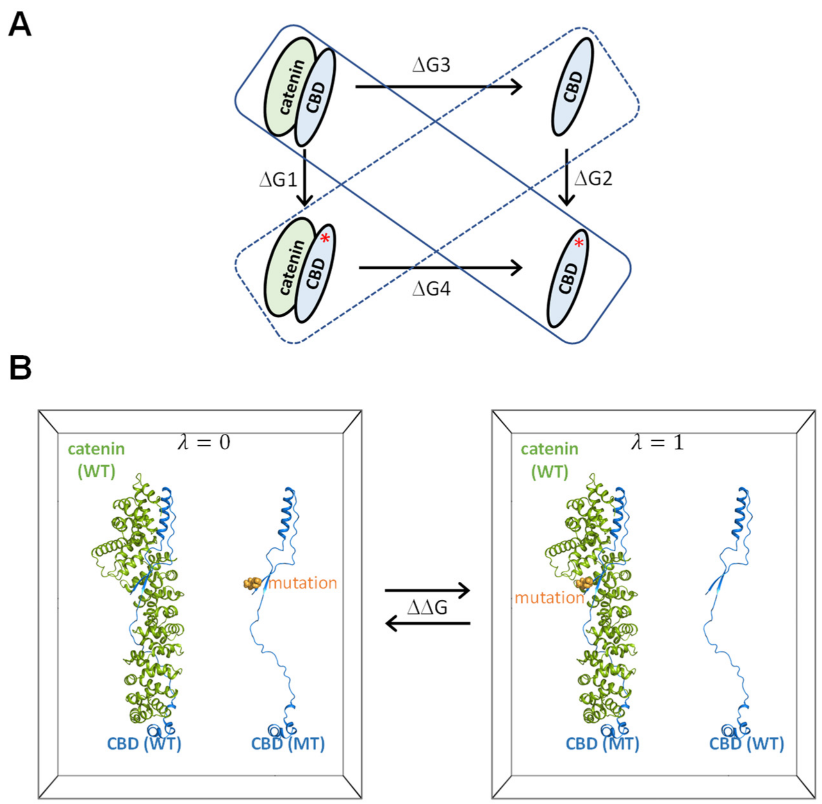


**Figure S1. Double-system/single-box setup.** (A) Two branches of a thermodynamic cycle are placed in one simulation box. The different boxes in the scheme are indicated by the solid ($\lambda = 0$) and broken ($\lambda = 1$) lines. (B) An example of a simulation setup for the catenin binding free energy calculation. The free energy difference corresponds to a double free energy difference: $\Delta\Delta G = \Delta G1 - \Delta G2$.

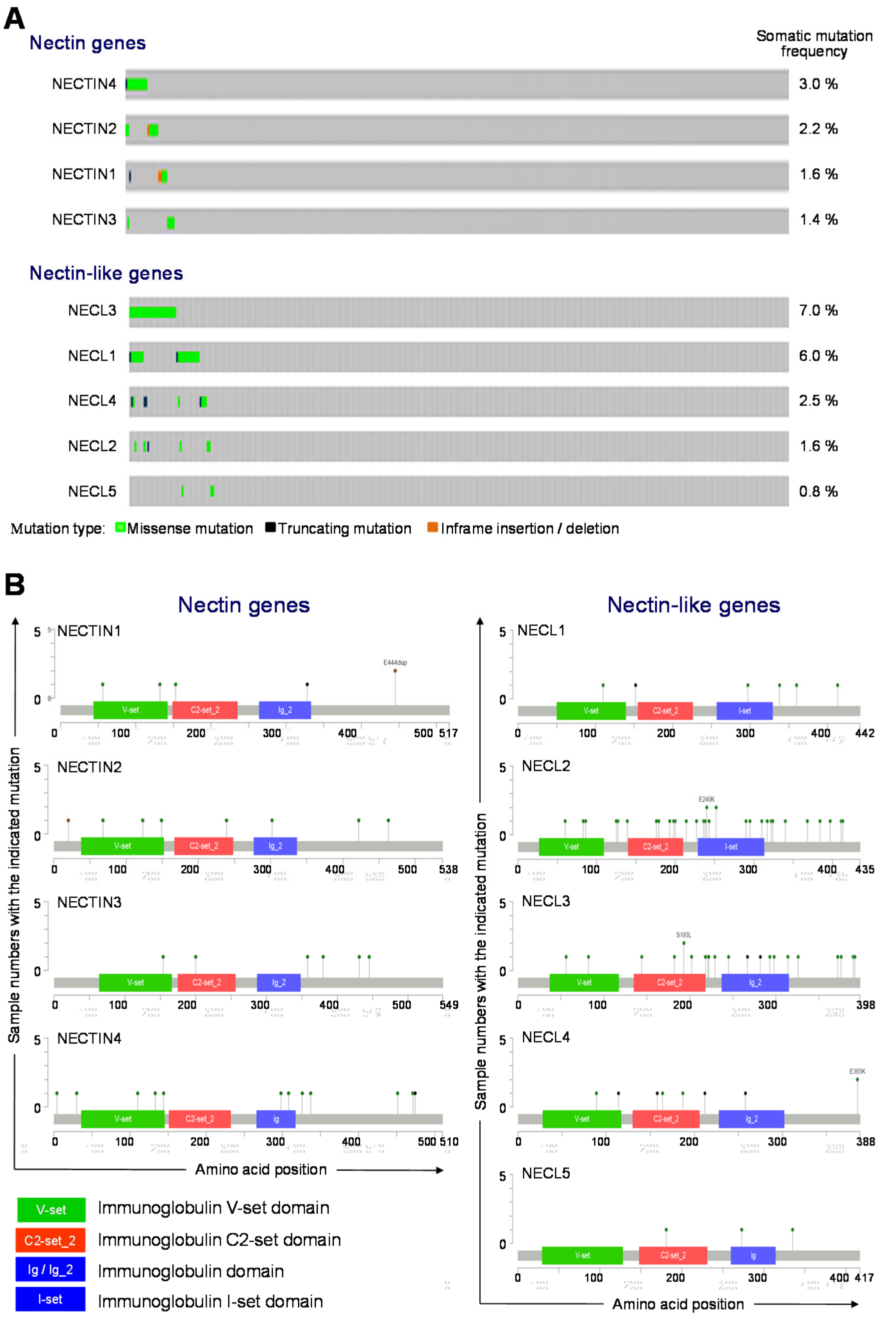


**Figure S2. Mutations in nectin and nectin-like genes in skin cutaneous melanoma.** (A) Mutation frequencies and (B) mutational profiles of nectin and nectin-like genes in skin cutaneous melanoma were analyzed by using NGS data from the TCGA cohort (TCGA, PanCancer Atlas; n = 448).

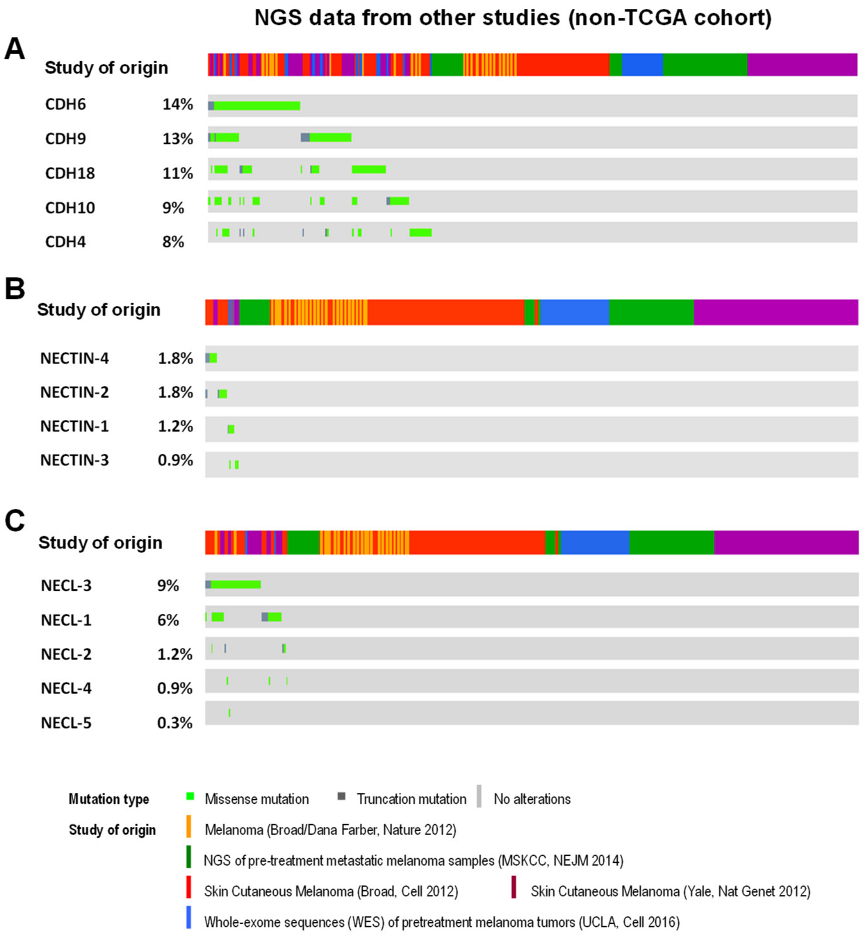


**Figure S3. Validation of mutation frequencies in key adherens junction (AJ) genes in non-TCGA cohort.** Mutation frequencies of key AJ genes, including (A) top-five highly-mutated cadherin genes identified in TCGA cohort, (B) nectin and (C) nectin-like genes, were analyzed by using NGS data from non-TCGA cohort (n = 396). The non-TCGA cohort include patients from Broad/Dana Farber (n = 26, Nature 2012), MSKCC (n = 64, NEJM 2014), Broad (n = 121, Cell 2012), Yale (n = 147, Nat Genet 2012), and UCLA (n = 38, Cell 2016).

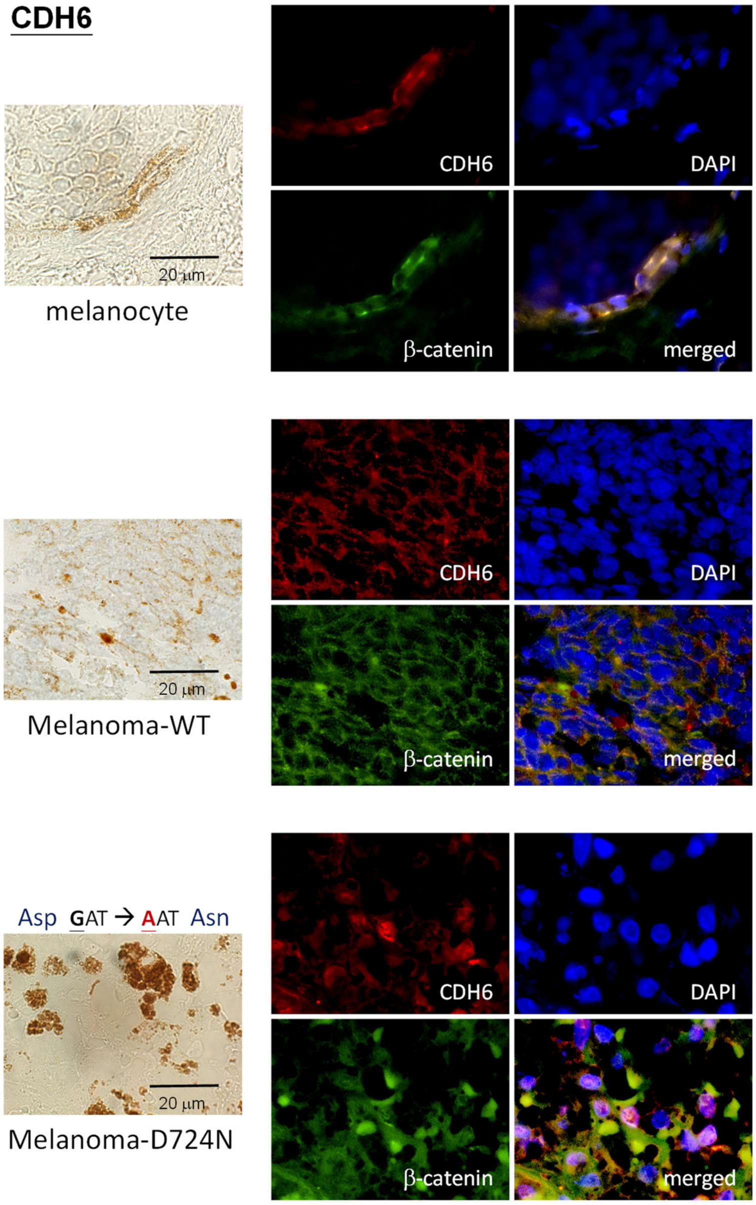


**Figure S4. Dual-color immunofluorescence staining of CDH6 and β-catenin on melanoma tissues from Taiwanese patients.** Tissue sections were prepared from Taiwanese patients with melanoma. Dual-color immunofluorescence staining, red for CDH6 and green for β-catenin, was performed on melanocyte from normal skin epithelium counterpart (*upper*), melanoma with wild type CDHs (*middle*), and melanoma with D724N mutation (*lower*). Cell nuclei were counterstained with DAPI.

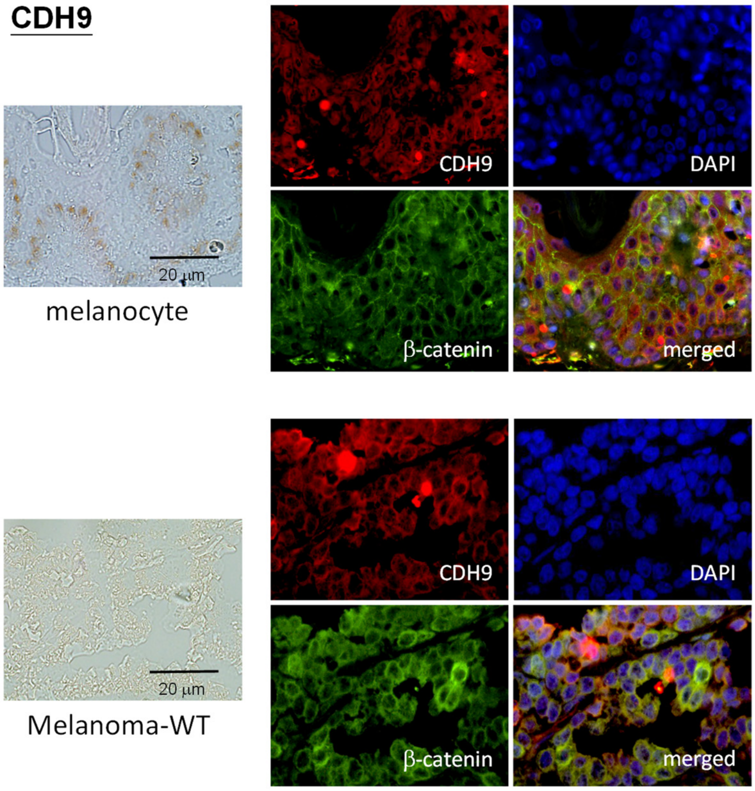


No mutation was detected in the intracellular domain of CDH9 in Taiwanese patients.

**Figure S5. Dual-color immunofluorescence staining of CDH9 and β-catenin on melanoma tissues from Taiwanese patients.** Tissue sections were prepared from Taiwanese patients with melanoma. Dual-color immunofluorescence staining, red for CDH9 and green for β-catenin, was performed on melanocyte from normal skin epithelium counterpart (*upper*), melanoma with wild type CDHs (*middle*). Cell nuclei were counterstained with DAPI.

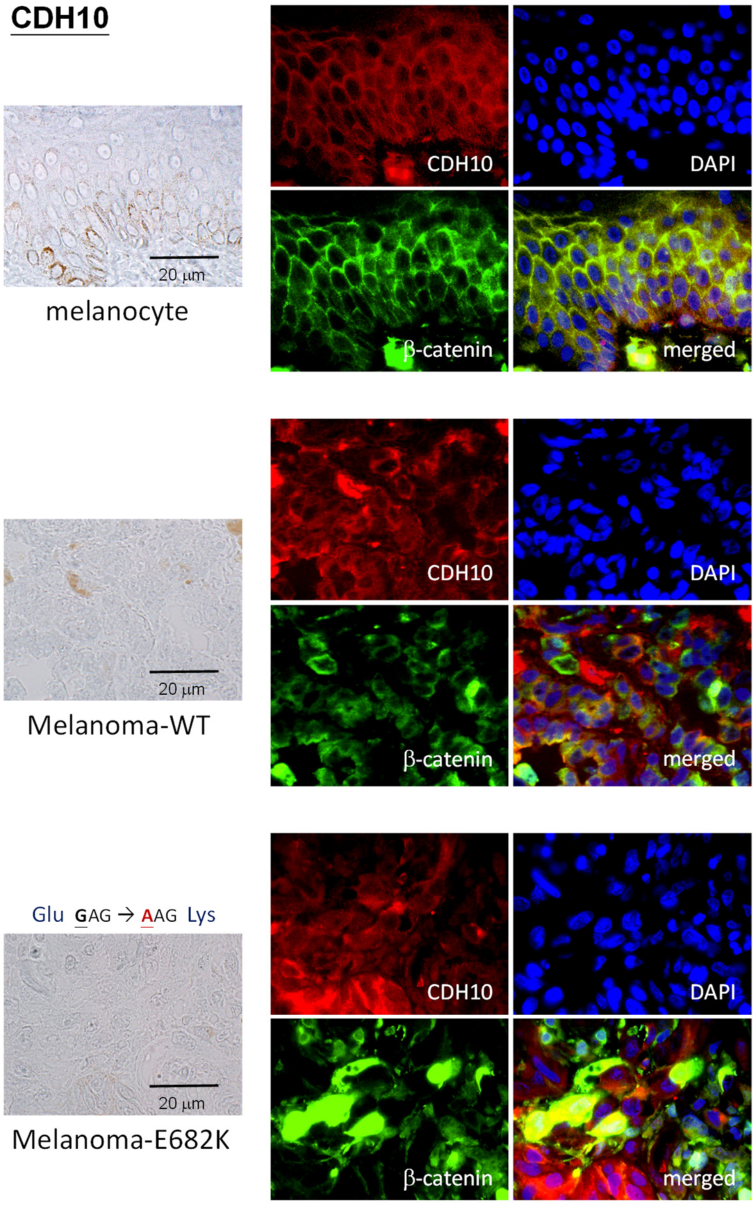


**Figure S6. Dual-color immunofluorescence staining of CDH10 and β-catenin on melanoma tissues from Taiwanese patients.** Tissue sections were prepared from Taiwanese patients with melanoma. Dual-color immunofluorescence staining, red for CDH10 and green for β-catenin, was performed on melanocyte from normal skin epithelium counterpart (*upper*), melanoma with wild type CDHs (*middle*), and melanoma with E682K mutation (*lower*). Cell nuclei were counterstained with DAPI.

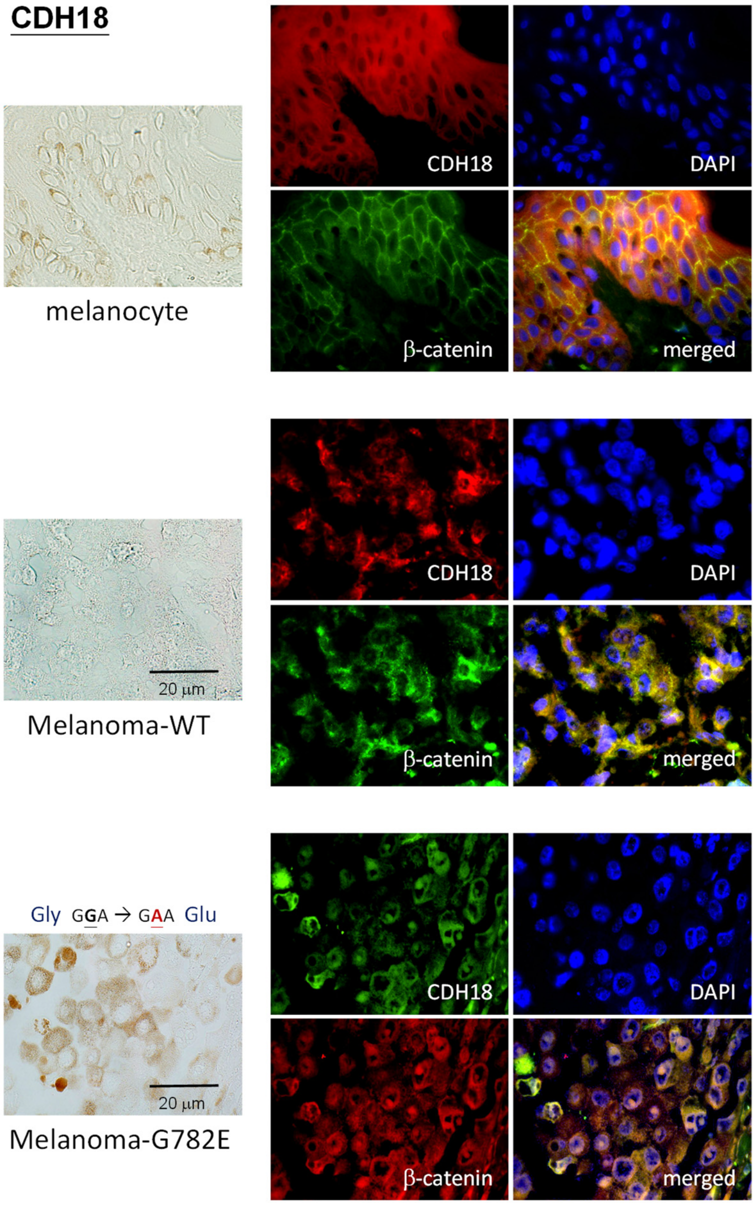


**Figure S7. Dual-color immunofluorescence staining of CDH18 and β-catenin on melanoma tissues from Taiwanese patients.** Tissue sections were prepared from Taiwanese patients with melanoma. Dual-color immunofluorescence staining, red for CDH18 and green for β-catenin, was performed on melanocyte from normal skin epithelium counterpart (*upper*), melanoma with wild type CDHs (*middle*), and melanoma with G782E mutation (*lower*). Cell nuclei were counterstained with DAPI.

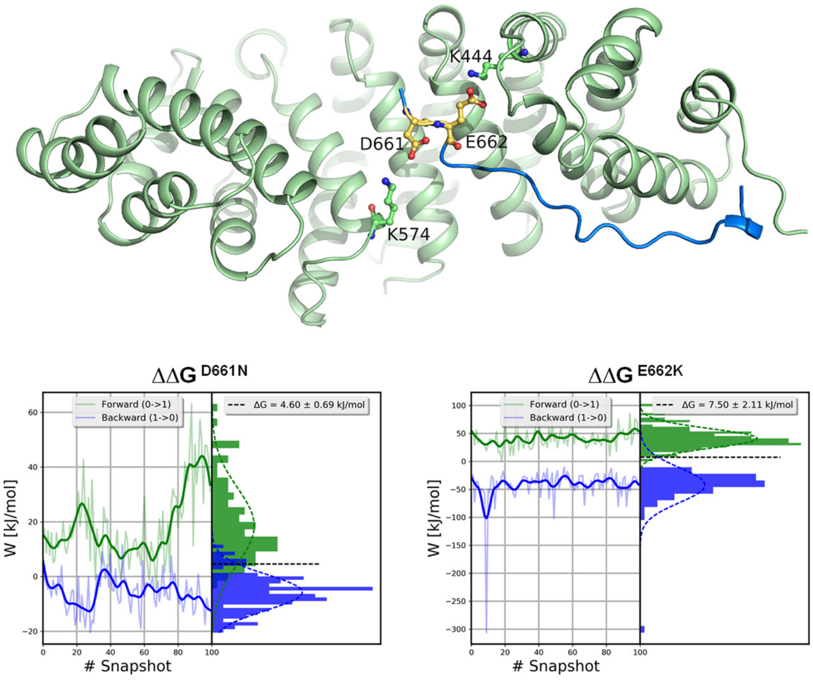


**Figure S8. The predicted CDH6/p120-catenin (δ-catenin) complex structures and their binding free energy differences (ΔΔ*G*) between WT and MT CDH6.** (A) The predicted structure of JMD (residues 660-677, in blue) in complex with p120-catenin (δ-catenin, in green) was generated by using the MODELLER program. The known mutated sites, D661 and D662, on the JMD (in orange) were found to interact with K444 and K574 on the p120-catenin, respectively. (B) A double-system/single-box setup was used to estimate the binding free energy differences (ΔΔ*G*) caused by mutations.

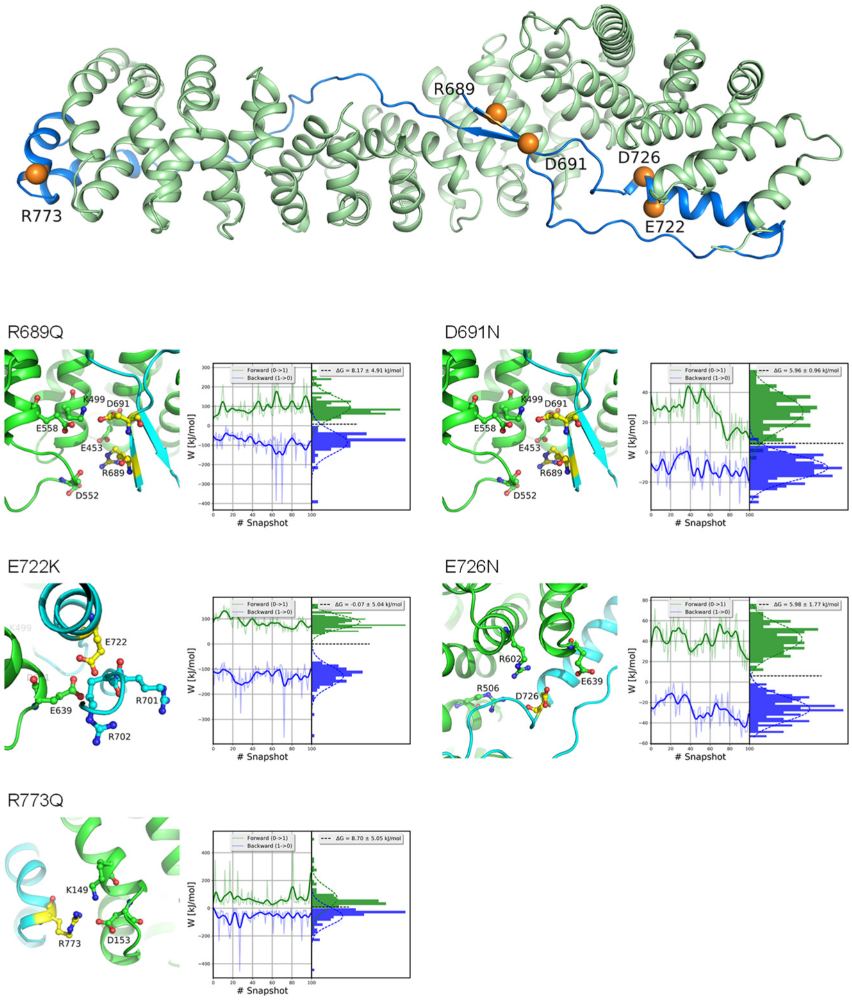


**Figure S9. The predicted CDH6/β-catenin complex structures and their binding free energy differences (ΔΔ*G*) between WT and MT CDH6.** (A) The predicted structure of CBD (residues 687-784, in blue) in complex with β-catenin (green) was generated by using MODELLER program. The known mutated sites were highlighted in orange spheres. A double-system/single-box setup was used to estimate the binding free energy differences (ΔΔ*G*) caused by (B) R689Q, (C) D691N, (D) E722K, (F) E726N, and (G) R773Q substitutions. Cartoon representations indicated the binding interfaces between mutated residues on CBD (yellow) and the interacting residues on β-catenin.

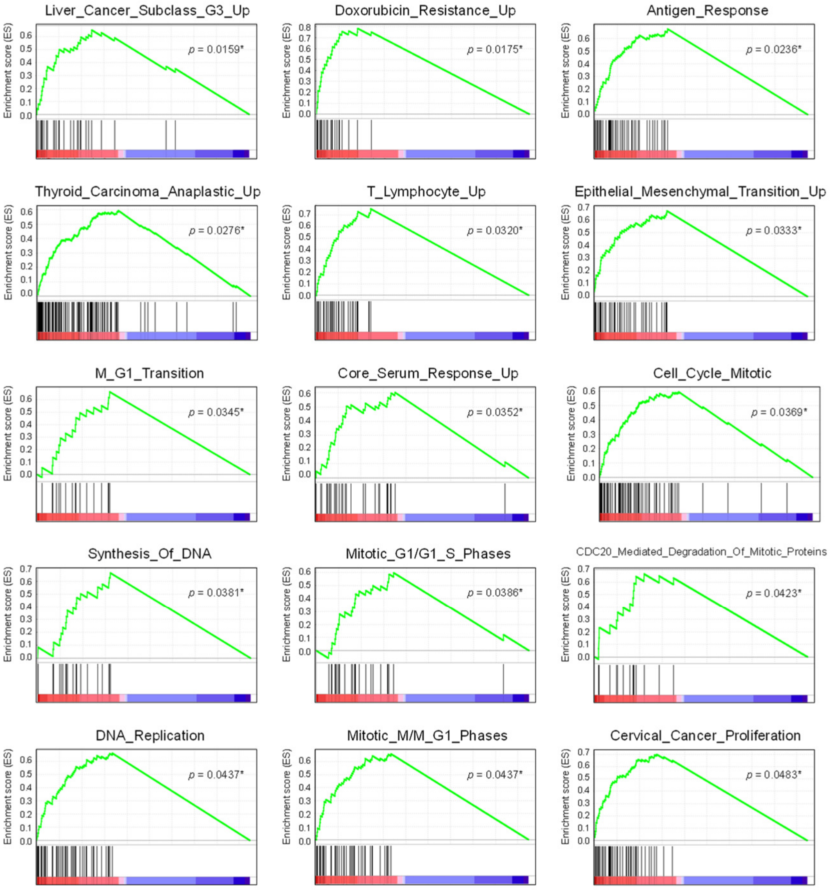


**Figure S10. Reactomes/pathways associated with mutations in top-four highly mutated cadherin (core CDHs) genes in skin cutaneous melanoma.** Gene set enrichment analysis (GSEA) was performed using mRNA expression data from the TCGA group (TCGA, PanCancer Atlas; n = 448). Patients without mutations in any cadherin genes were utilized as the controls. Concurrent up-regulated reactomes with *p*-values less than 0.05 were shown here. Two pathways, Antigen Response and T-Lymphocyte Up, were also shown in Fig. 7C.

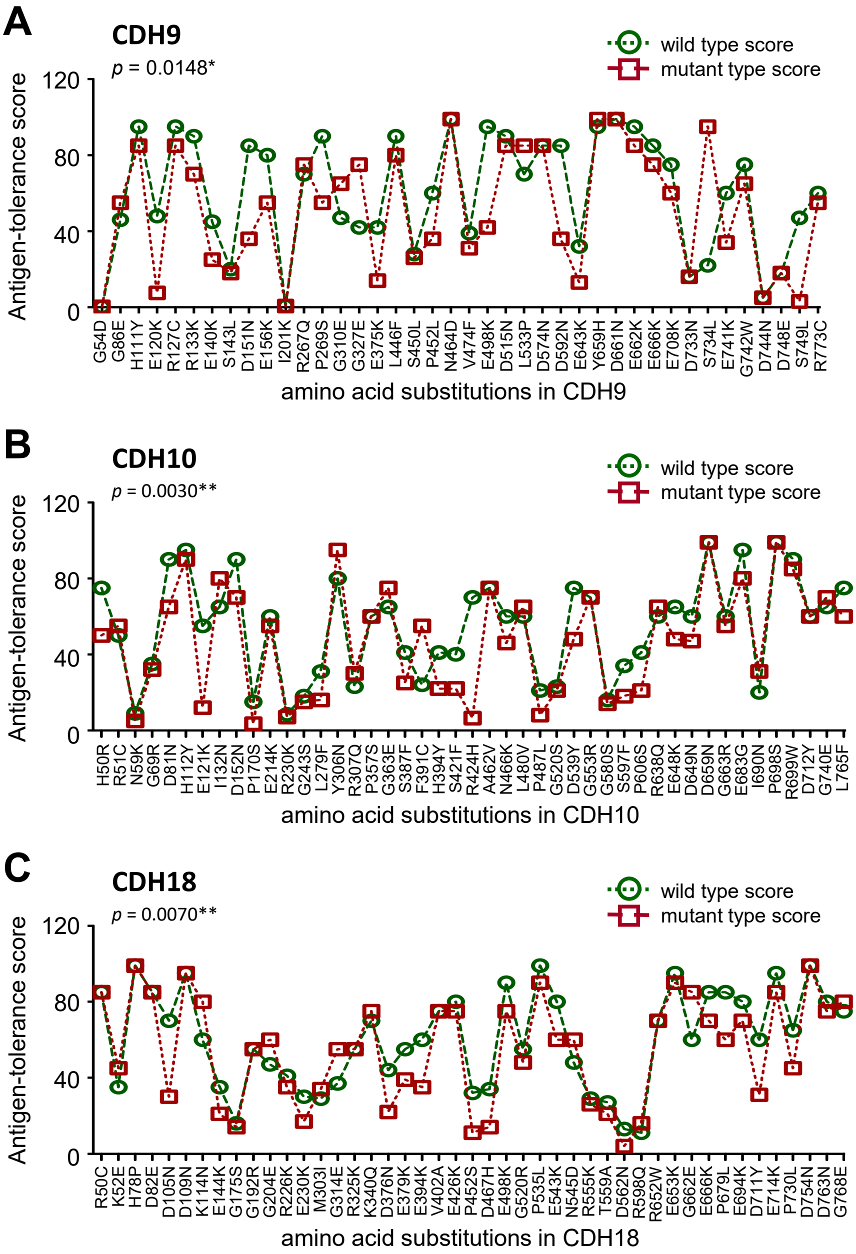


**Figure S11. Neo-antigen potentials of mutations in core CDH genes in skin cutaneous melanoma.** Antigen-tolerance scores of known mutations in the extracellular domain of (A) CDH9; (B) CDH10; and (C) CDH18 were analyzed by using NetMHCpan algorithm and compared with the scores with wild type sequence. Substitutions with lower scores indicate higher neo-antigen potentials. Two-way ANOVA was utilized to compare the differences between wild type and mutant type. Statistical significance: *, $p < 0.05$; **, $p < 0.01$.

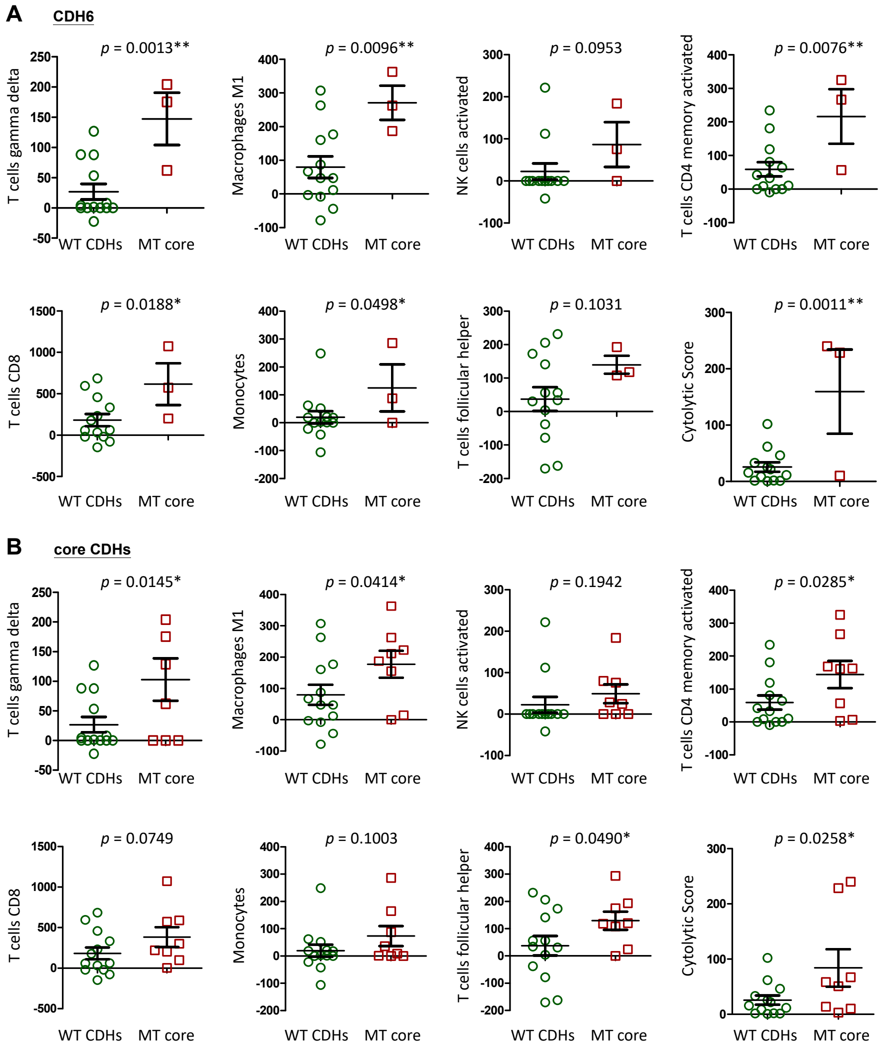


**Figure S12. Changes of tumor microenvironments in skin cutaneous melanoma by mutations in core CDH genes.** Microenvironment immune types were analyzed in tumors with mutations in (A) *CDH6* or (B) core CDH genes, and compared with the data in tumors with wild type CDHs. The immune scores include γδT cells, M1 macrophages, activated NK cells, activated CD4+ memory T cells, $CD8^+$ T cells, monocytes, follicular helper T cells and cytolytic score.
